# Corrected Super-Resolution Microscopy Enables Nanoscale Imaging of Autofluorescent Lung Macrophages

**DOI:** 10.1016/j.bpj.2020.10.041

**Published:** 2020-11-18

**Authors:** Ashley R. Ambrose, Susanne Dechantsreiter, Rajesh Shah, M. Angeles Montero, Anne Marie Quinn, Edith M. Hessel, Soren Beinke, Gillian M. Tannahill, Daniel M. Davis

**Affiliations:** 1 The Lydia Becker Institute of Immunology and Inflammation, Faculty of Biology, Medicine and Health, University of Manchester, Manchester, United Kingdom; 2 Department of Cardiothoracic Surgery, Manchester University NHS Foundation Trust, Manchester, United Kingdom; 3 Cellular Pathology, Wythenshawe Hospital, Manchester University NHS Foundation Trust, Manchester, United Kingdom; 4 Department of Anatomic Pathology, University Hospital Galway, Galway, Ireland; 5 GSK, Stevenage, United Kingdom

## Abstract

Observing the cell surface and underlying cytoskeleton at nanoscale resolution using super-resolution microscopy has enabled many insights into cell signaling and function. However, the nanoscale dynamics of tissue-specific immune cells have been relatively little studied. Tissue macrophages, for example, are highly autofluorescent, severely limiting the utility of light microscopy. Here, we report a correction technique to remove autofluorescent noise from stochastic optical reconstruction microscopy (STORM) data sets. Simulations and analysis of experimental data identified a moving median filter as an accurate and robust correction technique, which is widely applicable across challenging biological samples. Here, we used this method to visualize lung macrophages activated through Fc receptors by antibody-coated glass slides. Accurate, nanoscale quantification of macrophage morphology revealed that activation induced the formation of cellular protrusions tipped with MHC class I protein. These data are consistent with a role for lung macrophage protrusions in antigen presentation. Moreover, the tetraspanin protein CD81, known to mark extracellular vesicles, appeared in ring-shaped structures (mean diameter 93 ± 50 nm) at the surface of activated lung macrophages. Thus, a moving median filter correction technique allowed us to quantitatively analyze extracellular secretions and membrane structure in tissue-derived immune cells.

## Significance

Lung macrophages are the respiratory system’s first line of defense against pathogens. Light microscopy is a key tool for investigating immune cell biology, but lung macrophages are highly autofluorescent, restricting their analysis. Here, we developed and applied a correction technique for the super-resolution microscopy technique, STORM, using a moving median filter to separate autofluorescent background from selectively labeled signal of interest. We demonstrated that this technique accurately and robustly removed both simulated and real background autofluorescence. Application of this technique in the study of lung macrophages led to the accurate, nanoscale analysis of activation induced protrusions tipped with MHC class I protein and the tetraspanin protein, CD81, marking extracellular vesicle secretion. This technique will also enable super-resolution imaging of other autofluorescent samples.

## Introduction

Over the last two decades, light microscopy has moved beyond the diffraction limit to resolve structures on a nanometer scale using a range of different super-resolution techniques (1, 2, 3). Stochastic optical reconstruction microscopy (STORM) utilizes the stochastic blinking of fluorophores to allow the localization of individually labeled molecules. This has led to the observation of previously unresolved structures, such as periodic actin rings in axons (4), and changes in the nanoscale organization of immune cell receptors. For example, we recently demonstrated the segregation of the inhibitory receptor SIRP*α* from the activating receptor Fc*γ*RI on activated macrophages (5). This is suggestive of a potentially underappreciated spatial mechanism controlling the extent to which different receptor signals are integrated and demonstrates the importance of studying cellular architecture with super-resolution microscopy. Crucially, these and other observations were dependent on high-quality sample preparation, accurately calibrated instrumentation, high signal-to-noise fluorescence, and robust postprocessing (6).

Macrophages are the most abundant immune cell in a healthy human lung, comprising a heterogeneous population with specialized functions in the regulation of homeostasis and inflammation (7,8). Macrophages constantly communicate with other immune cells via cell-cell contacts, soluble factors, and extracellular vesicles (EVs). Recently, the role of EVs in lung injury has gained much attention, and EVs are thought to play a role in various inflammatory diseases, including chronic obstructive pulmonary disease (COPD) and acute respiratory distress syndrome (ARDS) (9, 10, 11, 12). Mechanisms and functions for EV release from the membrane of lung macrophages are an important scientific frontier to improve our understanding of tissue-specific immune responses and intercellular communication in general. Visualization of lung macrophages and EVs by super-resolution microscopy can facilitate quantitative analysis cell-by-cell, rather than a bulk analysis of vesicles obtained by ultracentrifugation.

Because lung macrophages are the primary immune cells to encounter environmental pathogens in the lung, they have an essential role in the presentation of antigen to in situ T cells, controlling T cell activation, function, and differentiation (13,14). The synapse formed with T cells involves a coordinated receptor organization that directly links to function (15). Therefore, understanding the nanoscale dynamics (9) of macrophage surface proteins is important to how they interact with other cells and initiate immune responses.

A major problem in using light microscopy to study lung macrophages is that they are highly autofluorescent. This unwanted “background” fluorescence obscures the signal of interest, which reduces image quality (16). Macrophages are well-known to be strongly autofluorescent, and this is even more pronounced with lung-derived macrophages at least in part because of their exposure to environmental contaminants such as cigarette smoke (17, 18, 19). Indeed, several studies report a correlation between autofluorescence and smoking history, although there are many other important factors. Perturbations of cellular metabolism like oxidation of pyridine nucleotides or the storage of aging pigments have also been reported to contribute to autofluorescence (20, 21, 22, 23). This high level of autofluorescence has been successfully utilized to identify macrophages, (24) but it has also been offered as an explanation for the unexpected, and now retracted, observation of an Foxp3+ population of mouse macrophages, demonstrating a confounding effect of autofluorescence on accurately interpreting results (25,26). Autofluorescence is particularly problematic when macrophages are isolated from the lungs of cancer patients, a high proportion of whom are smokers. An inability to differentiate background from a fluorescent label can have major implications and hampers a lot of research (18,27,28).

When utilizing most light-based microscopy methods, both the background autofluorescence and fluorescent signal appears simultaneously. Here, we report a correction technique that harnessed the conditions used for single-molecule localization microscopy (SMLM) to separate the two signals. Crucially in SMLM, the true fluorescence signal is distributed temporally as stochastic blinking events, whereas the background autofluorescence is relatively stable, appearing consistently throughout the acquisition. A variety of approaches were tested to identify what would best remove this constant background to yield corrected data sets. Analysis with simulated data identified that the most successful correction employed a moving median filter in agreement with previous attempts to improve STORM by reducing background (29). Application of this method enabled nanoscale images of lung macrophages. This led to detailed observations of MHC-class-I-protein-tipped protrusions on lung macrophages and nanoscale rings of CD81, indicative of EVs.

## Materials and Methods

### Cell lines

The epithelial cell line HEK293T/17, obtained from the American Tissue Culture Collection (Manassas, VA) was maintained in phenol-red free, high-glucose Dulbecco’s Modified Eagles Medium (Sigma-Aldrich, Gillingham, UK) supplemented with 10% FBS and 1% penicillin and streptomycin. Cells were detached using trypsin-EDTA and washed in media before plating (Thermo Fisher Scientific, Waltham, MA).

### Lung immune cell isolation

Human lung tissue was obtained from Manchester Allergy, Respiratory and Thoracic Surgery biobank, collected under National Health Service (NHS) Research Ethics Committee approval (15/NW/0409). Healthy tissue taken at least 10 cm from lung tumor resections was perfused with phosphate-buffered saline (PBS) using a 10-mL syringe with 0.8-mm needle (BD Biosciences, San Jose, CA). Immune cells were purified from the resulting perfusate by layering over Ficoll-Paque Plus (GE Healthcare, Chicago, IL) and centrifugation (400 × *g*, 35 min). Cells were collected from the layer directly above the Ficoll-Paque, washed twice, and counted. The perfusion process isolated all lung airway immune cells, including macrophages from alveolar spaces and larger airways.

### Sample preparation for imaging

5 × 10^4^ lung immune cells were plated for 15 min at 37°C on chambered glass coverslips (#1.5 LabTek II; Nunc, Roskilde, Denmark) coated with 0.01% poly-L-lysine (PLL) only, or PLL and 10 *μ*g/mL IgG (activating condition; both reagents from Sigma-Aldrich). Lung macrophages adhered and other immune cells were washed off with PBS at room temperature. Cells were fixed with 4% paraformaldehyde and PBS for 20 min, washed with PBS, blocked in 3% bovine serum albumin and PBS for 1 h, then stained for 1 h with specified antibodies diluted in blocking solution followed by 3 × 5 min PBS washes, all at room temperature. For intracellular staining, cells were permeabilized for 5 min with 0.1% Triton X-100 and PBS before blocking. Cells were stained with anti-*α*-tubulin monoclonal antibody (mAb) conjugated to Alexa Fluor 647 (AF647) (5 *μ*g/mL; clone YL1/2; Abcam, Cambridge, UK) or anti-CD81-AF647 mAb (10 *μ*g/mL; 5A6; BioLegend, San Diego, CA) or anti-MHC-I-AF647 or -AlexaFluor488 (AF488) (10 *μ*g/mL; W6/32; BioLegend, San Diego, CA), phalloidin-AF647 (3 U/mL), or wheat germ agglutinin (WGA)-AF647 or -AF488 (2 *μ*g/mL; Thermo Fisher Scientific). Antibodies were conjugated in-house, whereas dyes and stains were purchased already conjugated.

STORM imaging was performed with total internal reflection microscopy on a super-resolution ground state depletion (SR GSD) microscope (Leica Biosystems, Wetzlar, Germany) using a 160× 1.43 NA oil objective and a back-illuminated, electron-multiplying, charge-coupled device camera (iXon Ultra 897; Andor, Belfast, UK) with a 180 × 180 pixel cropped field of view utilizing a theoretical pixel size of 100 × 100 nm. Samples were temperature equilibrated on the stage after the addition of oxygen-scavenging buffer (560 *μ*g/mL glucose oxidase, 34 *μ*g/mL catalase, 1% *β*-mercaptoethanol, 25 mM glucose, and 5% glycerol (all Sigma-Aldrich), 25 mM HEPES and PBS (pH 8; 0.22-*μ*m filter sterilized; Thermo Fisher Scientific) for 30 min. Samples were then imaged with a 642-nm (2.1-kW/cm^2^) continuous wave laser using a 642 nm High Performance filter cube (405/10 and 642/10 excitation filters, a 649 dichroic and 710/100 emission filter) or a 488-nm (1.4-kW/cm^2^) continuous wave laser using a 488 nm High Performance filter cube (405/10 and 488/10 excitation filters, a 496 dichroic and 555/100 emission filter) for at least 9000 frames (11 ms). A 405-nm (30-mW) laser was used to reactivate AF647 and AF488 fluorescence (all lasers from MPB Communications, Montreal, Canada).

### Image processing

STORM images were analyzed using the ImageJ plugin, ThunderSTORM (30), as previously described (31). All data sets, including simulations, were analyzed using the same parameters. Predetection was carried out with a wavelet filer (B-spline; order 3 and scale 2), and approximate localization was calculated using local maxima (threshold; two standard deviations (SDs) of the first wavelet level). Subpixel localization was calculated using an integrated Gaussian point spread function with maximal likelihood fitting over a five-pixel radius. Filtering was not used for simulated data, but events with good localization precision from acquired data sets were retained using the following criteria: “intensity >500 and sigma >50 and sigma <200 and uncertainty_*xy* <30.” Molecules reblinking within 40 nm and 20 frames were merged together, and the sample drift was corrected for using cross correlation with three bins at 5× magnification. Localizations were then visualized using an average shifted histogram approach and magnified 10 × to realize a final effective pixel size of 10 × 10 nm.

Cross correlation was used because of high event density in all samples. To demonstrate the suitability of cross correlation, we compared it with the use of fiducial markers. For this, chambered glass coverslips were coated with 0.01% PLL containing 100-nm fluorescent nanodiamonds (20 *μ*g/mL; NDNV100nmHi; Adámas Nanotechnologies, Raleigh, NC). Cells were attached and stained with anti-CD81-AF647 mAb and imaged as previously described apart from three changes. Firstly, samples were not temperature equilibrated. Secondly, samples were imaged for 20,000 frames. Third, cells were selected to contain two or more peripheral nanodiamonds. Images were processed as previously described, with drift correction initially performed using the fiducial marker option in the ImageJ plugin, ThunderSTORM (settings; maximal distance = 40 nm, minimal marker visibility ratio = 0.5, and trajectory smoothing factor = 0.25). The data sets were then cropped to remove the nanodiamonds, and drift correction using cross correlation was performed (three bins at 5× magnification).

**Figure 5:**
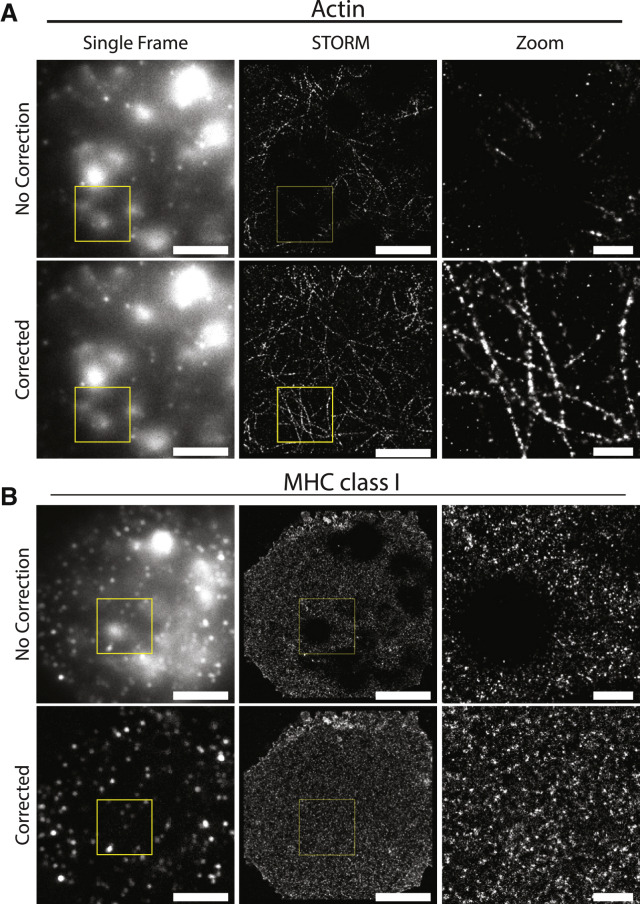
Moving median filter application enables super-resolution imaging of autofluorescent lung macrophages. (*A*) Acquired STORM data set of lung macrophages stained for filamentous actin with phalloidin-AF647 before and after correction. (*B*) Acquired STORM data set of lung macrophages stained with an AF647-conjugated anti-MHC-class-I mAb before and after correction. Scale bars, 5 and 1 *μ*m (zoom). Shown are both data sets imaged using a 642-nm continuous wave laser on a Leica SR GSD (Leica Biosystems).

**Figure 6:**
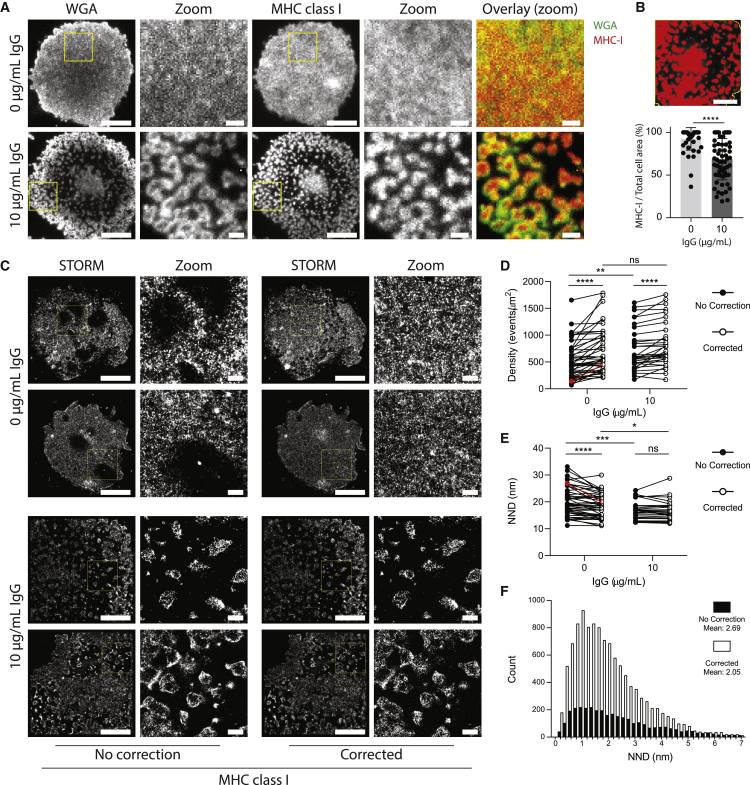
Lung macrophages form protrusions when activated through Fc*γ*RI. (*A*) Representative TIRF images of WGA (*left*) and MHC class I protein (*right*) on the surface of lung macrophages seeded onto PLL- (*top*) or PLL-and-IgG-coated (10 *μ*g/mL; *bottom*) glass slides for 15 min and stained with WGA-AF488 or anti-MHC-class-I mAb conjugated with AF647. Scale bars, 5 or 1 *μ*m (zoom). Cells imaged on a Leica SR GSD (Leica Biosystems) using 488- and 642-nm continuous wave lasers. (*B*) Percentage of area covered by staining for MHC class I protein relative to total cell area. Each dot represents a single cell, *n* = 4 individual donors and experiments, mean ± SD. (*C*) STORM images and zoom (5 × 5 *μ*m) of corrected and not corrected data sets showing MHC class I protein at the surface of human lung macrophages. Cells were incubated for 15 min on PLL- (*top*) or PLL-and-IgG-coated (10 *μ*g/mL; *bottom*) glass slides, fixed, and stained with anti-MHC-class-I mAb conjugated with AF647. Scale bars, 5 or 1 *μ*m (zoom). Cells imaged on a Leica SR GSD (Leica Biosystems) using a 642-nm continuous wave laser. (*D*) Density defined as the number of events per square micrometer of noncorrected and corrected STORM images in nonactivated and activated conditions. Each data point represents the density of an autofluorescent region (5 × 5 *μ*m) with a single region per cell. (*E*) NND of noncorrected and corrected STORM images in nonactivated (0 *μ*g/mL IgG; *left*) and activated (10 *μ*g/mL IgG; *right*) conditions. Each data point represents the mean NND of an autofluorescent region (5 × 5 *μ*m) with a single region per cell. (*D* and *E*) Five individual donors and experiments for 0 *μ*g/mL IgG and four for 10 *μ*g/mL. Data highlighted in red are the examples used in (*F*). (*F*) Histogram of all NND of one 5 × 5 *μ*m region in nonactivating condition: noncorrected versus corrected image. ^∗^*p* ≤ 0.05, ^∗∗^*p* ≤ 0.01, ^∗∗∗^*p* ≤ 0.001, ^∗∗∗∗^*p* ≤ 0.0001, by unpaired *t*-test, Mann-Whitney, or Wilcoxon test as appropriate. ns, not significant.

The difference in drift detected with the two methods after 20,000 frames was 2.86 ± 1.29 nm along the *x* axis and 3.71 ± 2.66 nm along the *y* axis (Fig. S1 *A*). When the two drift-corrected images were compared, they displayed a mean Mander’s overlap coefficient of 0.99 ± 0.013 (Table S6. Breakdown of Data, Related to Fig. S4, Table S7. Breakdown of Data, Related to Fig. S6 *B*), and this was seen at a range of event densities comparable with the lowest used in Figs. 5 and 6 (Fig. S1 *C*). Six example drift profiles, comparing cross correlation with fiducial markers, and their associated metrics are shown in Fig. S1 *D*. Overall, these data establish that cross correlation is suitable for correcting the drift occurring in the samples imaged here, with any small differences observed between fiducial markers and cross correlation being both beyond the resolution of the microscope and not impacting the biological findings.

### Background correction

Where described, background was removed using FIJI (32). STORM data sets underwent grouped z-projections using either average (mean) or median intensities across all or a specified number of frames. The resulting data were then resized with bicubic interpolation to match the number of frames in the original stack and to interpolate for changes between groups of frames in the varying gates. This produced a stack of calculated background, which was then subtracted from the original STORM data set to leave corrected data. This was carried out in batches using the following macro (example: moving median − 200-frame gate):

dir1 = getDirectory(“Choose Source Directory”);
dir2 = getDirectory(“Choose Destination Directory”);
list = getFileList(dir1);
setBatchMode(true);
for (i = 0; i < list.length; i++)
{
showProgress(i + 1, list.length);
open(dir1 + list[i]);
run(“Grouped Z Project...,” “projection = [Median] group = 200”);
run(“Size...,” “width = 180 height = 180 depth = 10,000 constrain average interpolation = Bicubic”);
imageCalculator(“Subtract create stack,” list[i], “MED_”+list[i]);
saveAs(“TIFF,” dir2 + list[i] + “_MovMedCorrected.tif”)
close(“^∗^”);
}

In addition to our ImageJ macro, we carried out background correction with two additional methods; firstly, using a temporal median filter implemented in Python and produced by Hoogendoorn et al. (29) and, secondly, a technique called HAWK that utilizes Haar wavelet kernels (HAWK) to separate dense fluorescent localizations from each other as well as background (33). These methods were used as described in their respective publications with the exception of using two temporal gates in the method published by Hoogendoorn et al., 101 frames as recommended and 201 frames to closely match the number of frames we determined as optimal.

### Data simulations

**Figure 1:**
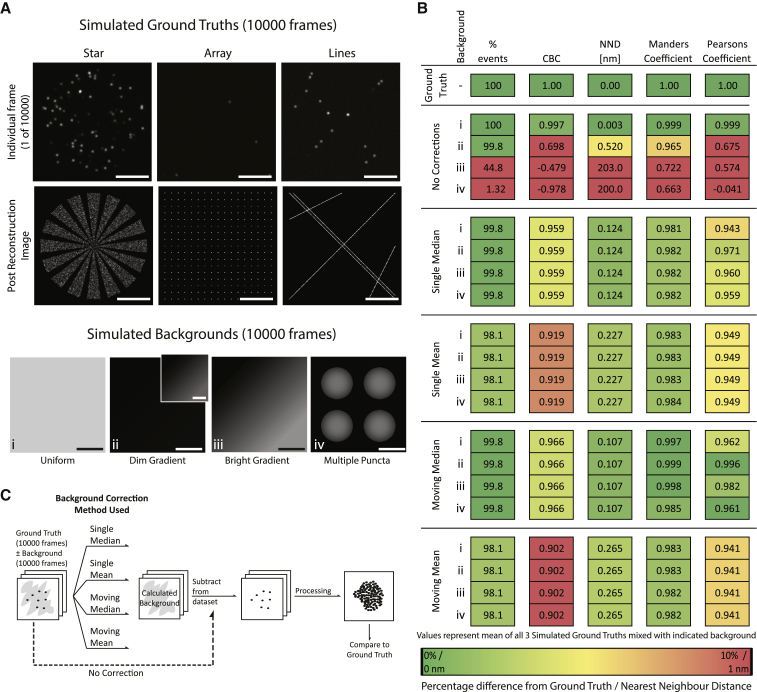
A comparison of different correction techniques in removing simulated backgrounds from simulated SMLM data. (*A*) Single frames and the final reconstructed STORM images from the three simulated ground truth (GT) data sets (*top*) and individual frames from the four simulated backgrounds (i–iv) used to test background correction techniques (dim gradient inset = scaled pixel intensity to highlight gradient). Scale bars, 5 *μ*m. (*B*) Quantitative assessment of different correction techniques. The three GTs were each combined with the four backgrounds (i–iv) to give 12 data sets and then processed (no correction) or corrected before processing with ThunderSTORM. The resulting images were assessed in relation to the GTs for the percent of events detected, coordinate-based colocalization (CBC), nearest neighbor distance (NND), Mander’s overlap, and Pearson’s correlation coefficient. Each value is a mean of the three GTs combined with one background (i–iv). (*C*) Workflow demonstrating the application of background correction techniques (single median, single mean, moving median, and moving mean corrections). The data set background was calculated and then subtracted from the original, yielding a corrected data set processed using ThunderSTORM.

Simulated ground truth data sets were generated using TestSTORM (34), a MATLAB plugin (The MathWorks, Natick, MA) that produced STORM data with bespoke characteristics. 10,000 frame data sets were generated to mimic the acquisition of data using the fluorescence label AF647 acquired with the Leica SR GSD microscope (Leica Biosystems) as closely as possible. This resulted in all simulations being 180 × 180 pixels, with a pixel size of 100 × 100 nm. These simulated data sets were shaped into predefined patterns (“star,” “array,” and “lines”) with varied event densities and intensities (Fig. S2). In addition, an average background level of 200 was applied to simulate camera noise via the addition of Poisson noise. To determine the nature of the localizations within the simulations, they were analyzed and visualized with ThunderSTORM as described above. These data were then used as the ground truth. Simulated backgrounds were generated using FIJI. Single images (Fig. 1 *A*) were transformed into stacks of 10,000 identical images or to mimic decaying autofluorescence a stack of five images with decreasing fluorescence intensity was resized to 10,000 images using bicubic interpolation (Fig. S2).

### Assessment of corrected images

Multiple metrics were used to describe the accuracy and precision of the correction technique, with all data sets being assessed in the same way relative to the specific ground truth. Specifically, data sets with or without corrections were compared with ground truths. Ground truths were based upon localizations detected through analysis of simulations in ThunderSTORM and were compared as follows. 1) Percentage of events, the total number of localizations detected was compared with relevant ground truths. 2) Coordinate-based colocalization (CBC), using ThunderSTORM, correlation between ground truths and matched data sets was calculated by comparing every ground truth localization and those within a 500-nm radius in the comparative data set. This value was displayed as a correlation ranging from −1 (completely disparate) through 0 (randomly colocalized) to 1 (perfectly correlated). Data are displayed as means or 0.1 binned histograms. 3) Nearest neighbor distance (NND), calculated alongside the CBC analysis, the distance between each ground truth event and the nearest event in the comparative data set was measured, shown as means or 0.1 nm binned histograms. 4) Mander’s overlap coefficient and 5) Pearson’s correlation coefficient, the co-occurrence and colocalization of events in the ground truth, versus no correction or corrected images were calculated using the Coloc2 plugin in FIJI.

### Assessment of membrane topology and MHC class I coverage

Lung macrophages on glass slides, coated with PLL or PLL with human IgG, were stained with WGA, and the total cell area was determined by the presence of fluorescence, manually selected, and measured in ImageJ. The area of MHC class I was calculated by thresholding using the default ImageJ method set to 75% of the highest gray value in each image. The proportion of MHC class I coverage was determined based on the area covered by MHC class I divided by total area calculated by WGA.

### Quantitative vesicle analysis

Quantitative vesicle analysis was performed on corrected STORM images of lung macrophages stained with anti-CD81-AF647. Vesicle density was determined by manually counting visible ring structures and then calculating density per square micrometer. Each ring structure diameter was measured using ImageJ.

### Data availability

**Figure 2:**
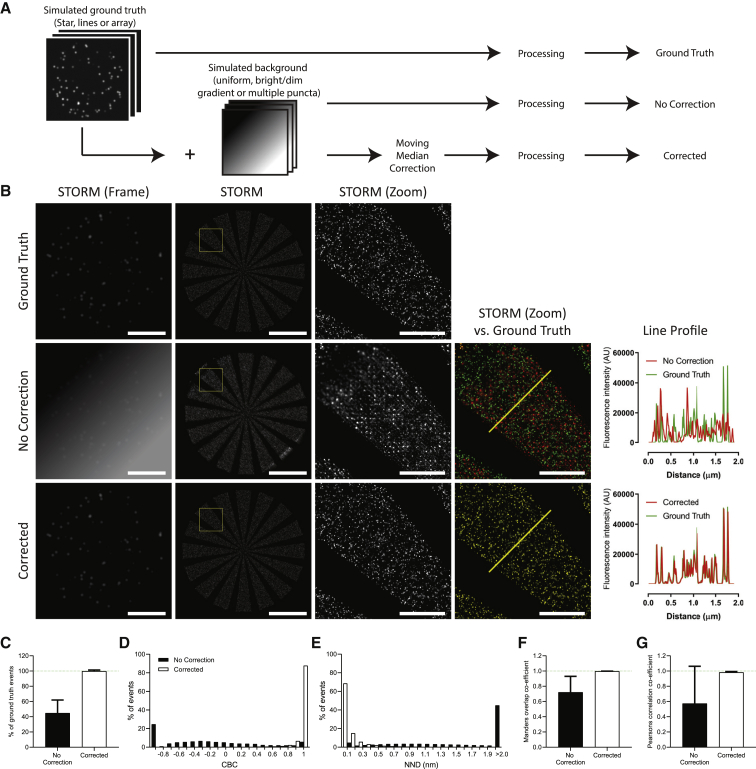
Moving median filter application before image processing successfully removes simulated backgrounds. (*A*) The simulated SMLM data sets were combined with simulated backgrounds or left alone and then corrected (moving median) or left uncorrected. (*B*) Comparison of the GT, no correction, and corrected data sets showing single frames, STORM images, zoom (3 × 3 *μ*m), and the overlay image (versus GT) with line profiles. Scale bars, 5 and 1 *μ*m (zoom). (*C*–*G*) Quantification of the three GTs mixed with the bright gradient background and no correction versus corrected images compared with the GTs. (*C*) Shown is the percentage of GT events detected. (*D*) CBC histogram. (*E*) NND distribution. (*F*) Mander’s overlap and (*G*) Pearson’s correlation coefficient. Error bars, ± SD.

**Figure 3:**
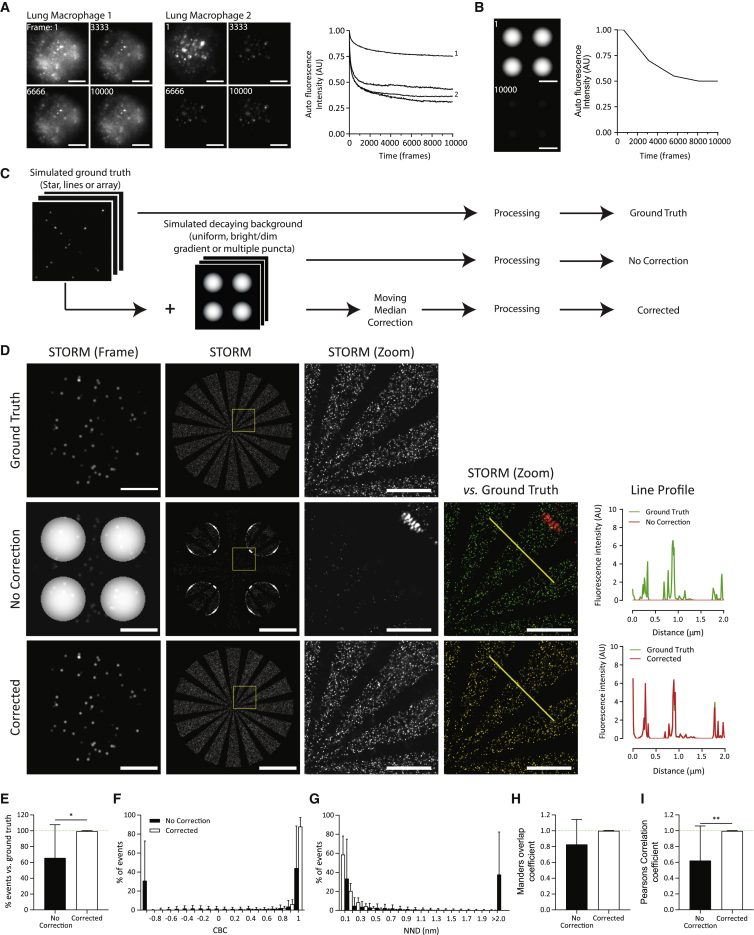
Application of a moving median correction successfully corrects simulated decaying backgrounds. (*A*) Characteristics of lung macrophages during STORM imaging using a 488-nm continuous wave laser. The fluorescent intensity of unstained lung macrophages was measured for 10,000 frames (*right*) and corresponding images from four different time points (*left*). (*B*) Simulated background with decaying intensity characteristics of lung macrophages. Fluorescence intensity (*right*) with images (*left*; corresponding frame indicated). (*C*) The simulated SMLM data sets were combined or not combined with simulated backgrounds with decaying intensity. The combined data set was corrected or not corrected before image processing and compared with the GT. (*D*) Single frame, STORM image, zoom (3 × 3 *μ*m), and overlay images with line profiles. Scale bars, 5 and 1 *μ*m (zoom). (*E*–*I*) Summary of all three GTs with all four backgrounds with intensity that slowly decays (from Fig. 1*A*) with and without correction relative to GT (*n* = 12). (*E*) Given is the percentage of events detected. (*F*) CBC histogram. (*G*) NND distribution. (*H*) Mander’s overlap and (*I*) Pearson’s correlation coefficient. Error bars, ±SD. ^∗^*p* ≤ 0.05, ^∗∗^*p* ≤ 0.01, by paired *t*-test.

**Figure 4:**
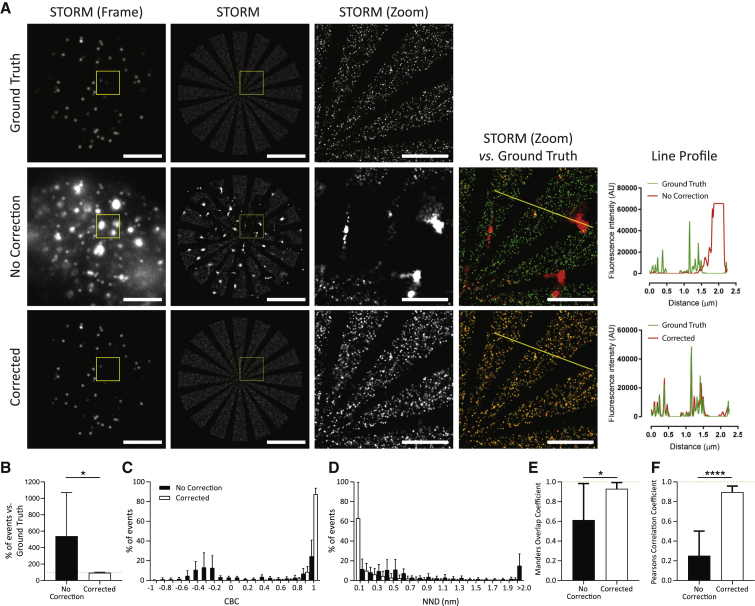
Correcting autofluorescence acquired from lung macrophages. The simulated SMLM data sets were left alone or combined with autofluorescent data sets from unstained lung macrophages (imaged on a Leica SR GSD (Leica Biosystems) using a 488-nm continuous wave laser) and were corrected or not corrected before image processing and comparison with GT. (*A*) Single frame, STORM reconstruction, zoom (3 × 3 *μ*m), and overlay images with line profiles. Scale bars, 5 and 1 *μ*m (zoom). (*B*–*F*) Quantification of the correction of all three GT with three separate unstained lung macrophage backgrounds (*n* = 9). (*B*) Percent of events detected normalized to the GT. (*C*) CBC histogram. (*D*) NND distribution. (*E*) Mander’s and (*F*) Pearson’s correlation coefficient. Error bars, ±SD. ^∗^*p* ≤ 0.05, ^∗∗∗∗^*p* ≤ 0.0001, by paired *t*-test.

All code and simulated data sets of background and ground truths are available online (https://doi.org/10.5281/zenodo.4226531). All individual values from assessment of simulated data in Figs. 1, 2, 3, 4, S3, S4, and S6 are available as Tables S1–S5, S6, and S7.

## Results

### A moving median filter successfully removes simulated background

Three separate ground truth data sets (referred to as star, array, and lines) with varying event density, arrangement, and coverage were each combined with four simulated backgrounds of differing intensities and structures. These backgrounds are referred to as uniform (i), dim gradient (ii), bright gradient (iii), and multiple puncta (iv) as shown in Fig. 1 *A*. To assess the accuracy and precision of comparative data sets, various parameters were measured; the percentage of events detected, CBC, NND, Mander’s overlap, and Pearson’s correlation of data sets, before and after correction, were relative to the ground truth.

First, the effect of adding simulated backgrounds to ground truths was assessed. Combining the uniform background (i) with the three ground truths had relatively little effect, whereas gradient backgrounds caused more pronounced problems (Fig. 1 *B*, no correction). The bright gradient background (iii), with intensity greater than simulated localizations, caused significant reductions in all metrics representing a low accuracy in replicating the three ground truths. The multiple puncta background (iv) resulted in the poorest reconstruction of the three ground truths, with only 1.3% of events detected, low Mander’s overlap coefficient (0.01), low correlation (Pearson’s = −0.171 and CBC = −0.500), and an NND of 75.7 nm.

The ability of four alternative image-processing techniques were compared for their ability to calculate and then subtract the simulated background from simulated SMLM data sets. Background correction was calculated in one of four ways: 1) measuring each pixel’s median intensity across all frames (single median), 2) measuring each pixel’s mean intensity across all frames (single mean), 3) measuring the median intensity across multiple, sequential 200-frame segments (moving median), or 4) measuring the mean intensity across multiple, sequential 200-frame segments (moving mean; Fig. 1 *C*). The data sets obtained from each of these correction methods were processed and compared with ground truths. This is presented in Fig. 1 *B* as a mean ± SD of the effect of each simulated background on the three ground truths (shown in Fig. 1 *A*), and individual values are presented in Table S1. All four correction methods were able to significantly increase the accuracy and precision of the data reconstruction, resulting in improved event detection, increased CBC, Mander’s and Pearson’s coefficients, and reduced NND. Furthermore, applying the correction methods to the three ground truth data sets without simulated backgrounds was not detrimental to reconstructions when single and moving median corrections were applied. However, single and moving mean corrections caused problems, resulting in reduced CBC and Pearson’s correlation values (Fig. S3; Table S5).

Between different correction methods, the use of a median-based correction performed better than those measuring mean pixel intensities (Fig. 1 *B*). Fluorescent blinking in SMLM produces pixel intensities that are stochastic outliers compared with the relatively uniform background. Because median values are more robust to outliers (35), it can be expected that the median corrections are best able to differentiate background and blinking events. This concept of robustness to outliers can be demonstrated via the “breakdown value” (36). A data set’s breakdown value is the maximal proportion of observations that can become outliers before yielding an incorrect result; this is 50% of observations for medians, but a single outlying observation is sufficient to produce a “false” mean. In the context of correcting STORM data, the estimation of autofluorescence intensity as a median value is significantly less susceptible to deviation in the presence of high intensity fluorescent events compared with estimating it as a mean.

By breaking the data set into multiple, sequential 200-frame sections, the moving mean was especially influenced by outliers because each blinking event represented a greater proportion of the signal, whereas these blinks remained outliers when the median was used. Therefore, the moving mean incorrectly estimated the background, resulting in especially poor event detection and localization (Fig. 1 *B*). Based on these data, the moving median correction technique was selected as the superior approach.

To test whether adjusting the number of frames used to calculate the moving median affected background removal and subsequent data set reconstruction, we used the same simulated data sets with medians calculated from 50, 100, 200, 500, 1000, and 2000 frames (Fig. S4; Table S6). This determined that the most accurate corrections were achieved with a 200-frame moving median and the worst with 50- and 2000-frame groupings.

To scrutinize the ability of a 200-frame moving median correction to remove background, an example data set is shown in Fig. 2 in which the Star ground truth was combined with the bright gradient background (Fig. 2 *A*). Corrected data were compared with the ground truth and data with no correction (Fig. 2 *B*). Example STORM frames demonstrate the challenge detecting individual events in the presence of the background, with dimmer events almost entirely lost. Distortion was especially pronounced when the gradient of background fluorescence was high. After correction, the image was almost perfectly restored, and this was true for all three ground truths mixed with the bright gradient background. There was an average of 99.8% of the number of ground truth events detected (no correction (NC) = 44.8%; Fig. 2 *C*), a mean CBC of 0.965 (NC = −0.479; Fig. 2 *D*), a Mander’s coefficient of 0.999 (NC = 0.721; Fig. 2 *F*), a Pearson’s coefficient of 0.982 (NC = 0.574; Fig. 2 *G*), and a mean NND reduced from 203.0 nm (NC) to 0.107 nm (Fig. 2 *E*). The individual data from each of the three ground truth data sets used in Fig. 2, *C*–*E* are detailed in Table S2. Thus, background correction using a moving median can restore the simulated SMLM data sets, resulting in accurate and precise event detection.

Because of the unique design of the three ground truths, there is a difference in event density for each: star = 68.8 events per frame, array = 3.8 events per frame, and lines = 19.9 events per frame. Investigating the individual values associated with the three ground truths indicates that in this context, event density is not a critical factor in the quality of the reconstruction.

### Removal of simulated autofluorescence

To apply this technique to autofluorescent lung macrophages, we first measured their autofluorescence in the absence of any staining under normal STORM imaging conditions. We observed that the autofluorescence reduced over time and that the rate of this decay continually decreased, plateauing after ∼5000 frames (Fig. 3 *A*). To mimic this, we altered simulated backgrounds (Fig. 1 *A*) to reduce at a continually slowing rate (Fig. 3 *B*). These four backgrounds were then each added to the three simulated ground truths, creating 12 separate simulations with decaying backgrounds, which were then corrected (Fig. 3, *C* and *D*).

Overall, the moving median background correction successfully identified the signal from the decaying background and improved every measured metric in all 12 simulations (Fig. 3, *E*–*I*; Table S3). The CBC of corrected data showed the biggest difference from the data with no correction, shifting from a mean of 0.16 ± 0.76 to 0.94 ± 0.03 (Fig. 3 *F*). In addition, the corrected data sets displayed strong Mander’s (Fig. 3 *H*; 0.998 ± 0.0007 vs. 0.827 ± 0.316) and Pearson’s coefficients (Fig. 3 *I*; 0.996 ± 0.001 vs. 0.58 ± 0.45) relative to the ground truth. These data demonstrate that backgrounds emulating autofluorescent macrophages can be corrected using a moving median filter.

### Simulated data containing autofluorescence from lung macrophages can be accurately restored

Simulated backgrounds represent an ideal scenario in which the autofluorescence decays consistently, but this does not exactly replicate the fluctuations observed with bona fide autofluorescent cells. Therefore, we next imaged unstained lung macrophage data sets of true cellular autofluorescence. These data were then added to the three simulated ground truths (those depicted in Fig. 1 *A*; star, array, and lines), to give a total of nine data sets. The example in Fig. 4 *A* shows that lung macrophage autofluorescence comprised an array of puncta in single STORM frames. During processing the centers of these large bright spots were frequently identified as fluorescent blinks. However, because of their large size, the detection of other events within the same space was prevented, causing small but concentrated areas of localizations surrounded by areas devoid of detectable events such as those seen in the zoomed data with no correction (Fig. 4 *A*, *middle row*). The addition of autofluorescence resulted in the detection of 539 ± 501% of the ground truth events (Fig. 4 *B*), reductions in CBC, Mander’s, and Pearson’s to −0.140, 0.613, and 0.252, respectively, and an increase in mean NND to 4.61 nm (Fig. 4, *C*–*F*; Table S4). However, after the application of the moving median correction, the number of events detected was 94.6 ± 7.2% (Fig. 4 *B*), and colocalization was significantly improved (CBC = 0.954, Mander’s = 0.918, Pearson’s = 0.885, and NND = 0.419 nm; Fig. 4, *C*–*F*). The background itself could also be calculated (Fig. S5). Together, this further demonstrates the ability of a moving median correction to identify and remove complex and decaying background autofluorescence, resulting in accurate and robust reconstructions of simulated data sets.

A temporal median filter has previously been applied to STORM acquisitions to estimate and remove background (29), but its effectiveness with regards to cellular autofluorescence has not been investigated. We therefore compared our approach with the temporal median filter from Hoogendoorn et al., which is implemented in Python. In addition, we compared a third technique that utilized HAWK analysis, a significantly different methodology that eliminates artifacts by enabling the separation of fluorophores because of their blinking behavior (33). For this comparison, we assessed the quality of correction on the 12 data sets in Fig. 1 *A* (simulated ground truth (GT) + simulated backgrounds with constant intensity), the 12 data sets in Fig. 3 (simulated GT + simulated backgrounds with slowing intensity decay), and the nine data sets from Fig. 4 (simulated GT + autofluorescent backgrounds). For the corrections, we used our method with a 200-frame grouping and the Hoogendoorn method with a 101-frame grouping, as recommended, and a 201-frame grouping as a direct comparison with the 200 frame grouping used here and the HAWK method. The results of this comparison are shown in Fig. S6.

All three methods produced corrected data sets that were significantly closer to the three ground truths regardless of background. When tested on simulated data with simulated backgrounds, our method and Hoogendoorn’s performed similarly (Fig. S6, *A*–*D*). The only observed difference was a lower Pearson’s coefficient for the Hoogendoorn technique (Fig. S6, *B* and *D*), which could be due to data being scaled by a mean fluorescence intensity profile before the application of the moving median filter. The techniques were also compared when the three ground truths were added to the three autofluorescent backgrounds, as used in Fig. 4. This revealed that our technique performed best in all metrics measured except for the Mander’s overlap coefficient, in which the method by Hoogendoorn et al. performed marginally better (Fig. S6, *E* and *F*).

Next, we combined acquired STORM data sets of HEK293T cells stained for filamentous actin with phalloidin or MHC class I protein with appropriate mAb in combination with the acquired autofluorescence from lung macrophages. Although these data are from two separate cell types, they allow us to accurately assess whether a moving median correction works in a situation in which the cellular “ground truth” and the cellular autofluorescence background are both well defined (Fig. S7). The addition of autofluorescence caused problems, as it did with simulated data, but this was corrected using the moving median. However, these data could not be quantified as in other figures because noise in the “ground truth” data were corrected in addition to the added autofluorescence. Altogether, both simulated and cell-derived data were affected by the addition of cellular autofluorescence, and the moving median correction was able to separate the signal from the noise.

### Removing autofluorescence from lung macrophages

Having proven the effectiveness of this method for background correction, autofluorescent lung macrophages were stained for filamentous actin using phalloidin (Fig. 5 *A*) or MHC class I protein using a mAb (Fig. 5 *B*) and imaged. Without correction, the autofluorescence was observed as large bright spots and an underlying haze that resulted in empty areas in processed super-resolution images. In contrast, these blank areas were removed in corrected images. Importantly, background correction allowed the visualization of filamentous actin structures, which could not be fully observed in the uncorrected image. This demonstrated that the background correction could be applied to highly autofluorescent samples, enabling the investigation of previously unusable samples, such as autofluorescent lung macrophages.

### Autofluorescent lung macrophages membrane topology and composition can be accurately quantified after correction

Immune cells from perfused human lung samples were plated onto glass slides coated with PLL or PLL with 10 *μ*g/mL human IgG for 15 min to trigger antibody-mediated activation via Fc receptors. The background correction allowed observation of the resultant model of a phagocytic synapse; macrophage membrane topology was visualized with WGA and MHC class I protein stained with a mAb. This revealed that cellular activation induced protrusions with MHC class I protein at the tips (Fig. 6 *A*). Here, the WGA stain and the shallow sample penetration with total internal reflection fluorescence (TIRF) microscopy imply that the changes observed for MHC class I are linked to a three-dimensional change in the cell surface topology rather than a reorganization of MHC class I into microclusters within the membrane. The area covered by MHC class I protein reduced from 92.4 ± 13.2 to 68.6 ± 24.2% of the total cell area after stimulation, demonstrating that this occurred across many cells (Fig. 6 *B*).

STORM was then used to quantify the nanoscale organization of MHC class I protein. Without background correction, large autofluorescent spots were observed that made visualization of the nanoscale organization impossible (Fig. 6 *C*, no correction). In activated conditions, less autofluorescence was evident, likely because of the altered membrane topology driving the cell body and autofluorescent particles away from the coverslip and beyond the TIRF evanescent field. To quantify this, we determined the event density and NND of corrected versus noncorrected images in activating and nonactivating conditions (Fig. 6, *D*–*F*). Correcting the images of nonactivated samples resulted in an increase in event density (Fig. 6 *D*) and decrease in NND (Fig. 6 *E*). In contrast, correction of the images from activated cells showed an increase in detected events without changing the NND, suggesting that the event distribution was largely unchanged.

The event density of nonactivating and activating conditions were significantly different until the correction was applied (Fig. 6 *D*; *p* = 0.0284 (NC) vs. *p* = 0.216 (corrected)). Similarly, the NND significantly decreased without correction, whereas the decrease after correction was much less significant (Fig. 6 *E*; *p* = 0.0011 (NC) vs. *p* = 0.0246 (corrected)). The effect of background correction was also clearly evident when comparing NND from one region (indicated in Fig. 6, *D* and *E* by *red symbols*) in a nonactivated cell (Fig. 6 *F*); many more events were detected in the corrected versus the noncorrected image (3366 vs. 11234), and the NND mean was reduced after correction (26.85 vs. 20.05). Overall, this clearly shows that the background correction is necessary for accurate quantitative analysis of STORM images from autofluorescent macrophages. Without this correction, accurate nanoscale organization of MHC class I protein at macrophage cell surfaces could not be inferred.

### Background correction of autofluorescent lung macrophages reveals nanometer-scale ring structures at the membrane

**Figure 7:**
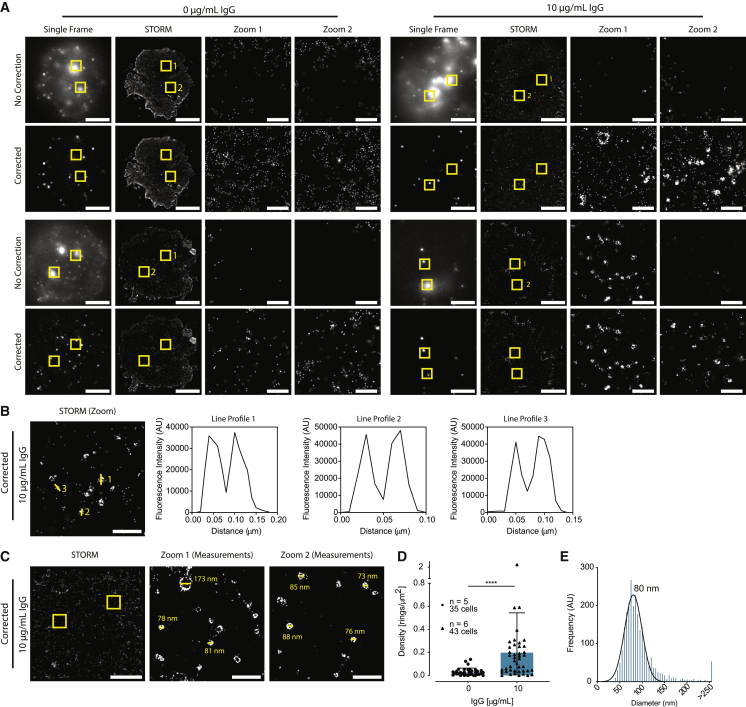
Observation of ring-shaped structures of CD81 in the membrane of IgG-activated lung macrophages. Lung macrophages were seeded onto glass slides coated with PLL (denoted as 0 *μ*g/mL) or PLL and 10 *μ*g/mL IgG for 15 min and stained with anti-CD81 mAb conjugated to AF647. All cells imaged on a Leica SR GSD (Leica Biosystems) using the 642-nm continuous wave laser. (*A*) Single frame, STORM, and zoom images (*boxed regions* in STORM images) of noncorrected and corrected images for nonactivated (0 *μ*g/mL) and activated (10 *μ*g/mL) conditions, two examples per condition. Scale bars, 5 *μ*m; zoom, 0.5 *μ*m. (*B*–*E*) Corrected images only. (*B*) Zoom image (2 × 2 *μ*m) of activated condition (10 *μ*g/mL IgG) with three line profiles of specific structures denoted 1, 2, and 3. Scale bars, 0.5 *μ*m. (*C*) STORM and corresponding zoom images with measurements of CD81 rings from the activated condition (10 *μ*g/mL IgG). Scale bars, 5 *μ*m; zoom, 0.5 *μ*m. (*D*) Density of ring structures per square micrometer. Each data point represents one cell. Bar indicates mean and error bars, SD. (*E*) Histogram of ring diameters of activated cells with fitted Gaussian. Mode is 80 nm; *n* = 5 (0 *μ*g/mL) or *n* = 6 (10 *μ*g/mL) individual donors and experiments. ^∗∗∗∗^*p* ≤ 0.0001, by unpaired *t*-test.

Lung macrophages were plated onto slides coated with PLL (nonactivating conditions) or PLL with 10 *μ*g/mL IgG (activated conditions) for 15 min and stained for the tetraspanin and EV-marker CD81 using a mAb conjugated with fluorescent dye AF647. In both activated and nonactivated conditions, images before correction showed bright spots of autofluorescence that resulted in blank areas in the reconstructed STORM image (Fig. 7 *A*, no correction). Upon correction, the specific stain in these regions was recovered (Fig. 7 *A*, corrected). In nonactivated conditions, CD81 showed a homogenous distribution with no structure clearly discernible. However, upon activation, the formation of ring-shaped structures was observed (Fig. 7 *A*, 10 *μ*g/mL IgG). In regions of high autofluorescence, these structures were completely obscured but could be observed and quantified after correction. This highlights the necessity of a correction method to accurately detect nanoscale structures at the surface of autofluorescent cells. In regions without autofluorescence, these structures are visible both before and after correction, demonstrating that they are not artifacts created by the correction method (Fig. 7 *A*, 10 *μ*g/mL IgG, second example, zoom 1).

An example region, shown in Fig. 7 *B*, highlights the ring-shaped structure of CD81 likely to be secreted vesicles. CD81-enriched structures were counted, and their diameter was measured (examples shown in Fig. 7 *C*). Few, if any, were clearly visible on unactivated cells, but their frequency increased dramatically upon activation (0.03 ± 0.03 compared with 0.20 ± 0.35 rings/*μ*m^2^; Fig. 7 *D*). The diameter of these structures ranged from 50 to 250 nm on activated cells (Fig. 7 *E*). A Gaussian fitting identified the mode at 80 ± 19 nm, whereas the mean diameter was 93 ± 50 nm. Together, this demonstrates that the median correction method allows for accurate detection and investigation of specific structures, likely secreted vesicles, at the surface of autofluorescent lung macrophages.

## Discussion

Autofluorescence is a limiting factor for the application of light-based microscopy in a variety of samples, and many methods have previously been suggested to eliminate this unwanted signal. Chemical quenching (37,38) and laser bleaching before staining (39,40) have been attempted with limited success, and as here (Fig. 3 *A*), even 10,000 frames of high laser power cannot bleach the autofluorescence of lung macrophages. A laser wavelength alternative to that used for exciting fluorophores has been used to identify and correct for background (41). However, autofluorescence varies across channels and detectors. Imaging with far-red fluorophores is less susceptible to autofluorescent background (42), but this is limiting, and the background signal is not entirely diminished. Altogether, applying these methods to lung macrophages only partially reduced autofluorescence or induced artifacts. Here, we report a novel, to our knowledge, background correction method for SMLM data using a moving median, which enabled the imaging of highly autofluorescent human macrophages.

SMLM enables the differentiation of signal from background because of the different temporal signatures, intermittent stochastic blinks for signal, and constant background as noise. The application of a moving median filter to simulated and acquired data sets clearly demonstrated its robustness to remove a variety of background structures and isolate fluorophore blinks from autofluorescence. A variety of metrics were measured, establishing that this is a precise and robust correction. Critically, in areas with variance from the ground truth, changes were below the theoretical 20-nm resolution of STORM (1), with 99% of events in all corrected reconstructions localizing within 10 nm of their expected location (combined data from Figs. 3, 4, and S7). These slight differences are likely attributable to a small amount of noise after correction (43).

Median filtering is already widely applied in a spatial manner to microscopy image processing and analysis, particularly for the robust removal of noise caused by outliers, whereas temporal filters have been used to smooth data and aid cellular and particle tracking (44,45). In addition, background correction is incorporated into SMLM analysis packages (e.g., ThunderSTORM). However, as evidenced here, these corrections proved ineffective for structured, bright, and uneven backgrounds, such as those associated with autofluorescence. Hoogendoorn et al. previously implemented a more complex temporal median filter using Python (29) that has been applied in STORM analysis tools such as FIRESTORM (43) and the commercial software, Huygens Localizer (Scientific Volume Imaging, Hilversum, the Netherlands). This technique is more complicated relative to the use of the simple method tested here, and although it performs similarly with simulated data, its ability to correct for macrophage autofluorescence is less accurate. Overall, removing unwanted noise from SMLM data sets with a moving median correction implemented with an ImageJ macro is a simple and effective technique with wide applicability.

Here, we first utilized this technique to investigate lung macrophage membrane topology and the organization of MHC class I protein. Lung macrophages activated through Fc receptors formed protrusion-like structures. In general, cells form different types of protrusions with many different functions. For example, macrophages attach to serum-opsonized bacteria and surround them with membrane protrusions before phagocytosis (46). In addition, Junt et al. showed that specialized macrophages deliver antigens to B cell follicles through macrophage protrusions in lymph nodes (47). T cells may scan the surface of antigen-presenting cells, including macrophages, using T cell receptor accumulated in the tips of protrusions (48). This, coupled with our observation of MHC class I protein being accumulated in macrophage protrusions, suggests that the initiation of T cell activation by macrophages may occur via novel protrusion-to-protrusion interactions. This type of interaction is somewhat reminiscent of filapodial bridges and nascent nanotube structures (49). Clearly, improved microscopy methods have a major role to play in elucidating the precise sequence of events that lead to immune cell synapse formation and activation (50,51).

These observations in STORM were only possible after application of the correction method. The density of MHC class I protein at the surface of lung macrophages did not change upon activation, but the mean NND decreased slightly. This meant that MHC class I proteins were packed more densely in activating conditions, whereas the overall number of molecules remained the same. Denser clusters have previously been shown to have the potential to induce stronger signals (31,52,53), suggesting a functional significance of dense MHC class I packing to activate T cells. However, it is not clear how many MHC class I proteins within a cluster would be likely to have an antigenic peptide.

Further investigation of the membrane of lung macrophages led to the discovery of nanoscale rings of CD81 that appeared upon cellular activation. Importantly, this observation was only possible when the moving median correction was applied because these structures were obscured in highly autofluorescent regions. Postcorrection, a detailed and accurate quantitative assessment was possible. Here, we found that their density increased from 0.031 ± 0.033 to 0.20 ± 0.35 rings/*μ*m^2^ upon macrophage activation via Fc receptors. The mean diameter of the ring structures was 93 ± 50 nm, whereas a Gaussian fitting identified the mode at 80 ± 19 nm.

CD81 is part of the tetraspanin family and commonly used as a marker of EVs (54,55). EVs are a heterogeneous group of membrane particles secreted by all cell types that are classically divided into apoptotic bodies, microvesicles, or exosomes according to their subcellular origin, size, and constitution (56,57). Although characteristics of the different EV types overlap, small vesicles with diameters ranging from 40 to 160 nm and of endosomal origin are considered to be exosomes (58). Thus, the observed rings of CD81 at the surface of activated lung macrophages are likely to be exosomes. Indeed, 94% of all ring structures observed were within the expected size range of exosomes. STORM has been used in the past to study EVs (59,60). However, in all these previous studies, EVs were isolated before imaging. The isolation of EVs is a harsh process that can damage them (61). These data establish that STORM can study EV secretion in situ. We have recently studied secretions from human natural killer cells that are not autofluorescent, and uncovered a surprising level of heterogeneity (62). Now, using the correction technique described here, autofluorescent immune cells, which have previously been extremely challenging to investigate, can also be scrutinized. Overall, these findings highlight the complexity of intercellular communication and how super-resolution imaging has an ever-increasing role to play in understanding this.

## Author Contributions

A.R.A. and S.D. designed, performed, and analyzed the experiments. A.R.A., S.D., and D.M.D. conceived the project and wrote the manuscript. E.M.H., S.B., and G.M.T. helped to conceive the work. R.S., M.A.M., and A.M.Q. helped obtain and prepare the lung tissue samples.

## Supporting Material

Document S1. Figs. S1–S7 and Tables S1–S5

Table S6. Breakdown of Data, Related to Fig. S4

Table S7. Breakdown of Data, Related to Fig. S6

Document S2. Article plus Supporting Material
