# Supplementary material for: Corrected Super-Resolution Microscopy Enables Nanoscale Imaging of Autofluorescent Lung Macrophages
Source: Biophys J. 2020 Nov 18;119(12):2403–17. doi: 10.1016/j.bpj.2020.10.041 (PMC7822748; doi:10.1016/j.bpj.2020.10.041)
Supplement: Document S1. Figs. S1–S7 and Tables S1–S5 [file mmc1.pdf]

Biophysical Journal, Volume *119*

Supplemental Information

Corrected Super-Resolution Microscopy Enables Nanoscale Imaging of Autofluorescent Lung Macrophages

Ashley R. Ambrose, Susanne Dechantsreiter, Rajesh Shah, M. Angeles Montero, Anne Marie Quinn, Edith M. Hessel, Soren Beinke, Gillian M. Tannahill, and Daniel M. Davis

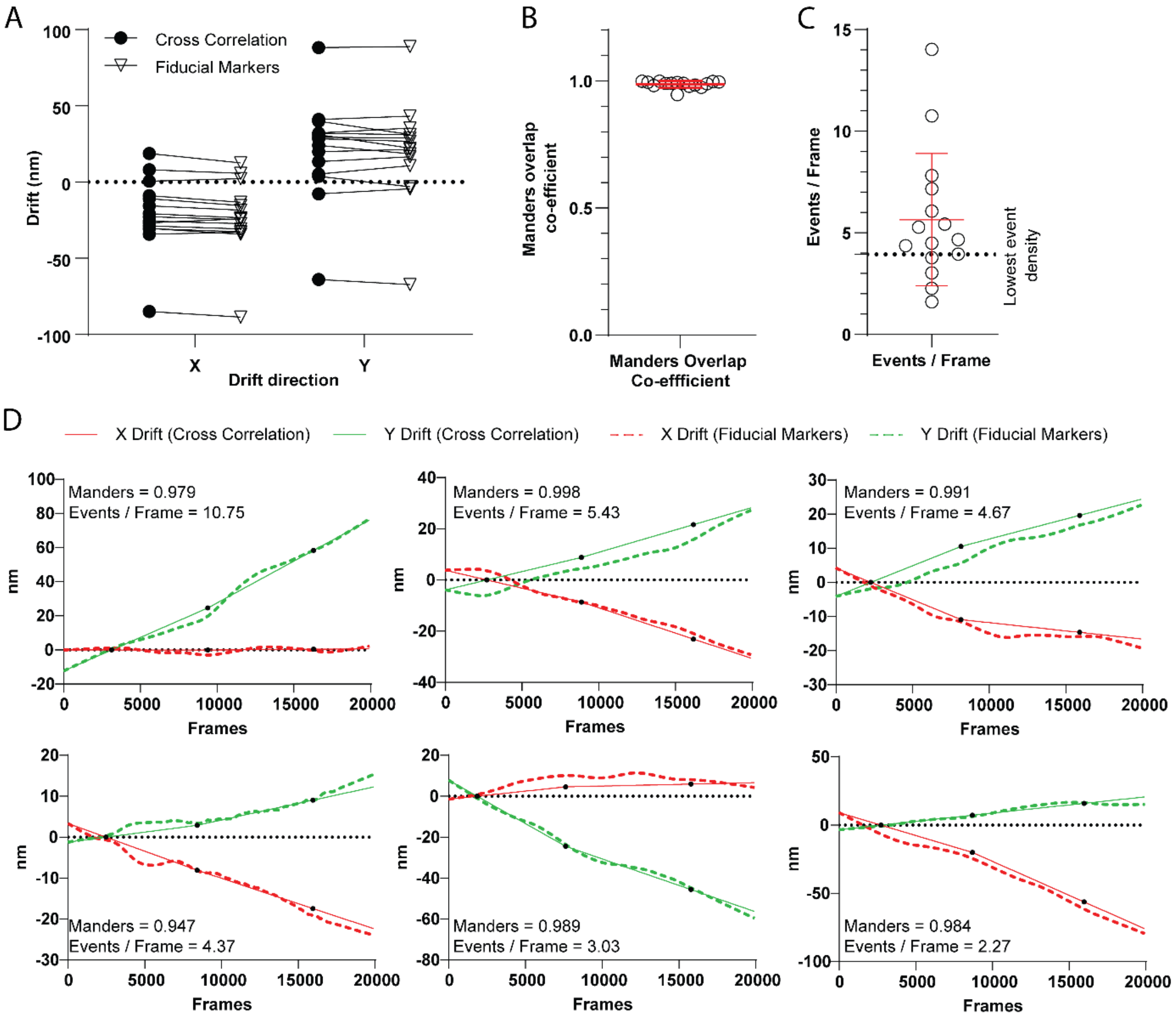

Supplementary Figure 1: Drift correction by cross correlation is comparable to drift correction by fiducial markers. *Cells were plated onto slides coated with PLL and 20µg/mL fluorescent nanodiamonds, stained with αCD81-AF647 and imaged, without temperature equilibration to trigger increased drift, using STORM for 20000 frames. Acquired datasets were then processed in ThunderSTORM and then the drift was corrected using either fiducial markers or, after removal of fiducial marker fluorescence, by cross correlation. A) A comparison of the total measured drift for each dataset over 20000 frames in the X or Y direction comparing cross correlation to fiducial markers (n=15). B) Manders overlap coefficient of the final images following drift correction with cross correlation or fiducial markers (n=15). C) Events / frame of imaged samples with an indicator of the lowest event density of any sample used in Figure 5 or 6 (n=15). D) Example drift correction traces from 6 separate samples comparing cross correlation (solid lines) and fiducial markers (dotted lines).*

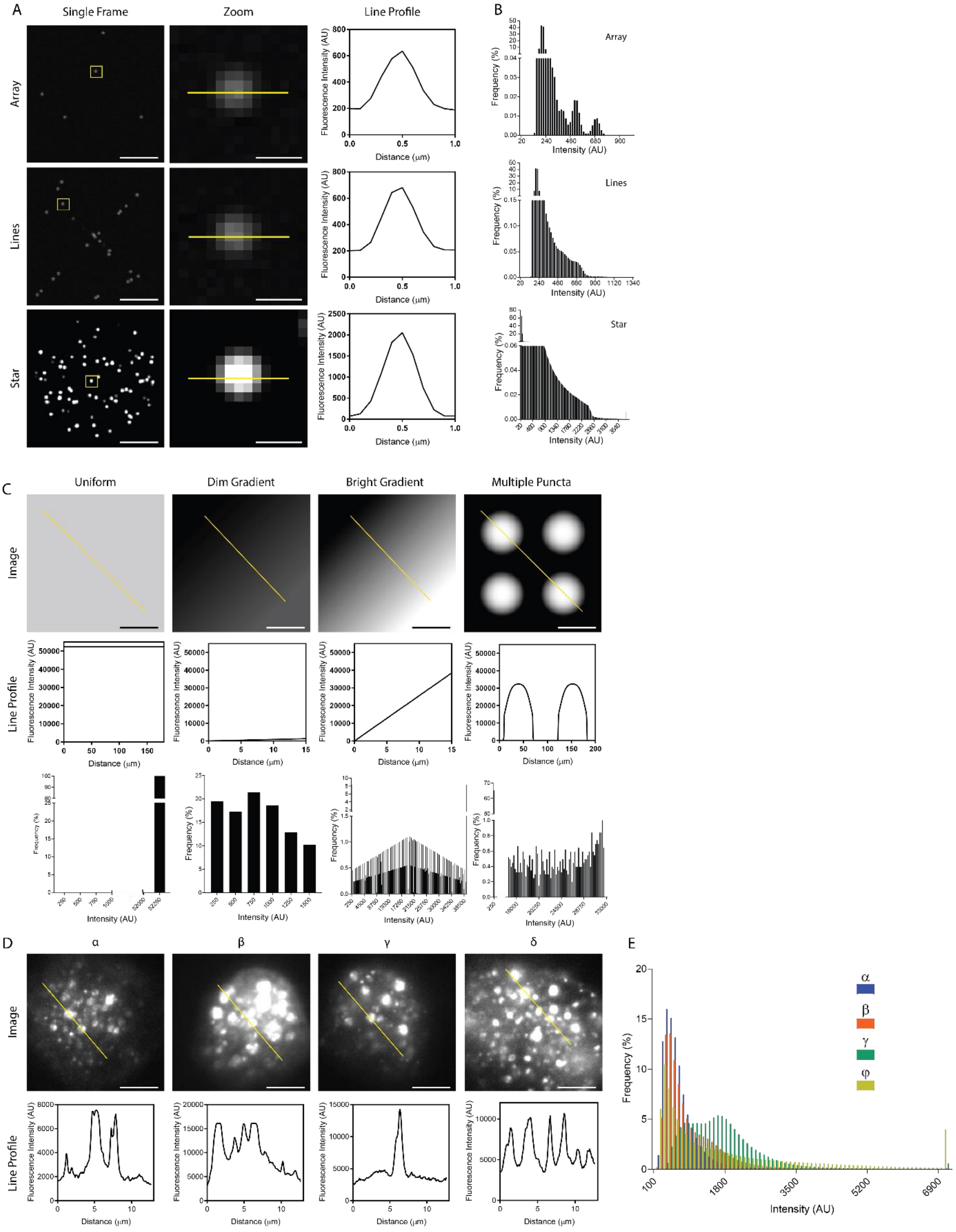

Supplementary Figure 2: Intensity profiles of ground truths (GT) and backgrounds. A) *Single frame and zoomed regions (1.5 x 1.5 μm) with line profiles of the 3 different GT used in this paper. Scale bars: 5 μm, Zoom 0.5 μm.* ***B)*** *Histogram of intensities of all pixels from each dataset (10,000 frames).* ***C)*** *Single frame (top) with line profile (middle) and histogram of intensities of all pixels in the dataset (bottom) of simulated backgrounds with constant intensities over 10,000 frames.* ***D)*** *Lung macrophages were plated onto PLL-coated glass slides for 15 min, fixed and STORM datasets of unstained cells were acquired. Single frame (top) with line profile (bottom) of the auto-fluorescence from lung macrophages.* ***E)*** *Histogram of intensities of all pixels for datasets in (D).*

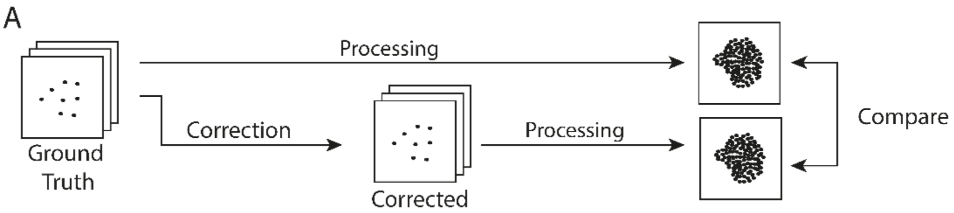

B

	% of events	CBC	NND (nm)	Manders Coefficient	Pearsons Coefficient
**Ground Truth**	100	1.00	0.00	1.00	1.00
**Single Median**	101	0.966	0.28	0.999	0.990
**Single Mean**	98.5	0.954	0.41	0.999	0.897
**Moving Median**	100	0.985	0.26	1.000	0.997
**Moving Mean**	98.4	0.952	0.44	0.999	0.887

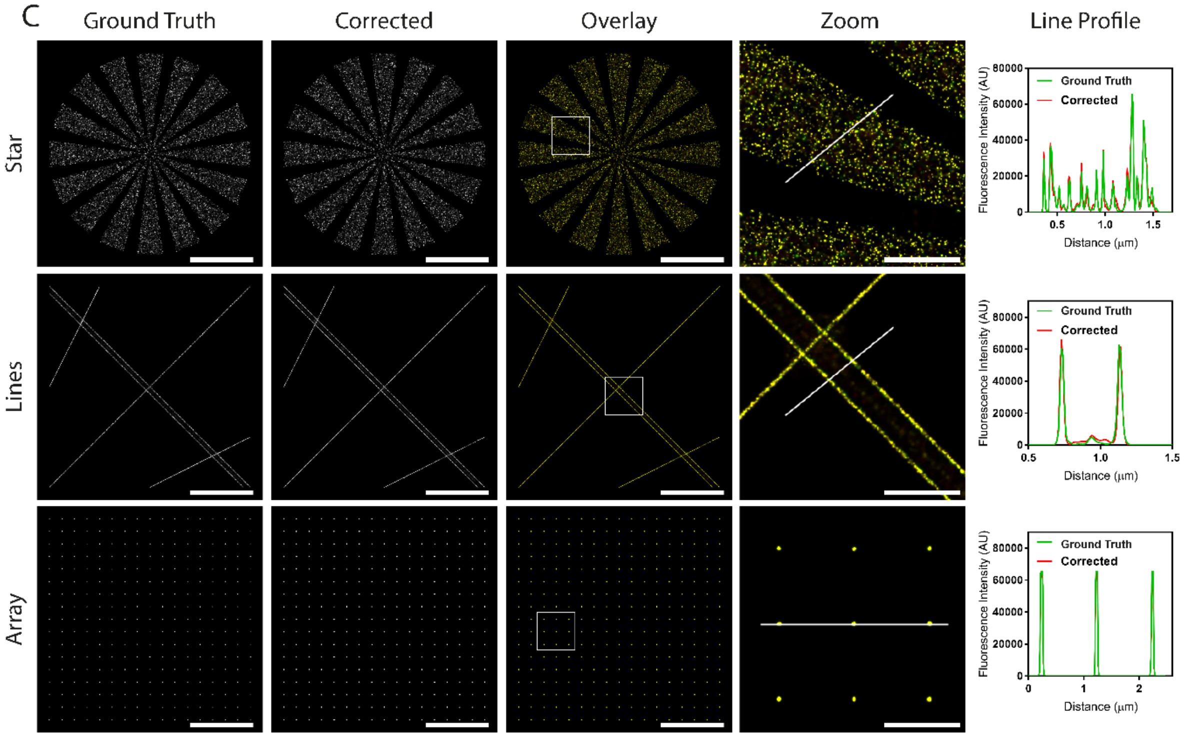

Supplementary Figure 3: Median background corrections are not detrimental to datasets without background. A) *Ground Truth (GT) datasets were processed following different corrections (single median, single mean, moving median and moving mean) and then compared to the processed GT without correction.* ***B)*** *Datasets were compared to the uncorrected GT to assess the number of events detected, coordinate based co-localisation (CBC), nearest neighbour distance (NND) and co-occurrence and co-localisation via Manders and Pearsons correlation respectively. Full data in Supplementary Table 5.* ***C)*** *Worked examples of 3 separate GT corrected with moving median, showing the processed STORM images, overlays with the uncorrected GT and corresponding line profiles. Scale bars: 5 μm and 1 μm (zoom).*

A — Constant Background

	Background	% of events	CBC	NND (nm)	Manders Overlap Co-efficient	Pearsons Correlation Co-efficient
Ground Truth	-	100	1.00	0.00	1.00	1.00
No Corrections	i	100	0.997	0.03	0.999	0.999
No Corrections	ii	99.8	0.698	0.52	0.965	0.675
No Corrections	iii	44.8	-0.479	203	0.722	0.574
No Corrections	iv	1.32	-0.978	200	0.663	-0.041
50	-	98.9	0.911	0.183	0.999	0.997
50	i	98.9	0.911	0.183	0.997	0.942
50	ii	98.9	0.911	0.183	0.998	0.993
50	iii	98.9	0.911	0.183	0.990	0.940
50	iv	98.9	0.911	0.183	0.833	0.896
100	-	99.8	0.952	0.129	0.997	0.942
100	i	99.8	0.953	0.129	0.996	0.935
100	ii	99.8	0.953	0.129	0.997	0.942
100	iii	99.8	0.953	0.129	0.997	0.939
100	iv	99.8	0.953	0.129	0.997	0.937
200	-	99.8	0.965	0.107	0.999	0.996
200	i	99.8	0.965	0.107	0.997	0.962
200	ii	99.8	0.965	0.107	0.999	0.996
200	iii	99.8	0.965	0.107	0.998	0.982
200	iv	99.8	0.965	0.107	0.985	0.962
500	-	99.8	0.968	0.099	0.999	0.997
500	i	99.8	0.968	0.099	0.997	0.927
500	ii	99.8	0.968	0.099	0.998	0.987
500	iii	99.8	0.968	0.099	0.971	0.873
500	iv	99.8	0.968	0.099	0.860	0.868
1000	-	99.8	0.968	0.096	0.999	0.985
1000	i	99.8	0.968	0.096	0.997	0.964
1000	ii	99.8	0.968	0.096	0.999	0.985
1000	iii	99.8	0.968	0.096	0.999	0.978
1000	iv	99.8	0.968	0.096	0.994	0.971
2000	-	99.8	0.969	0.094	0.999	0.997
2000	i	99.8	0.969	0.094	0.998	0.948
2000	ii	99.8	0.969	0.094	0.999	0.995
2000	iii	99.8	0.969	0.094	0.997	0.968
2000	iv	99.8	0.969	0.094	0.854	0.926

B — Decaying Background

	Background	% of events	CBC	NND (nm)	Manders Overlap Co-efficient	Pearsons Correlation Co-efficient
Ground Truth	-	100.0	1.000	0.000	1.000	1.000
No Correction	i	100	0.997	0.030	0.999	0.999
No Correction	ii	100	0.839	0.370	0.985	0.810
No Correction	iii	57.4	-0.336	45.0	0.688	0.516
No Correction	iv	4.98	-0.836	118.5	0.634	-0.031
50	-	98.9	0.911	0.183	0.999	0.997
50	i	98.0	0.902	0.208	0.999	0.997
50	ii	98.9	0.911	0.138	0.999	0.997
50	iii	98.4	0.908	0.197	0.999	0.997
50	iv	98.7	0.907	0.208	0.999	0.997
100	-	99.8	0.952	0.129	0.997	0.942
100	i	98.8	0.928	0.183	0.997	0.942
100	ii	99.8	0.953	0.129	0.997	0.942
100	iii	99.2	0.944	0.156	0.997	0.942
100	iv	99.5	0.942	0.158	0.997	0.942
200	-	99.8	0.965	0.107	0.999	0.996
200	i	99.1	0.928	0.186	0.999	0.996
200	ii	99.8	0.966	0.109	0.999	0.996
200	iii	99.3	0.949	0.156	0.999	0.996
200	iv	99.4	0.944	0.159	0.999	0.996
500	-	99.8	0.968	0.987	0.999	0.997
500	i	99.3	0.920	0.200	0.999	0.997
500	ii	99.8	0.969	0.103	0.999	0.997
500	iii	99.4	0.942	0.175	0.999	0.997
500	iv	109	0.923	0.189	0.999	0.997
1000	-	99.8	0.968	0.096	0.999	0.985
1000	i	99.2	0.917	0.204	0.999	0.985
1000	ii	99.8	0.968	0.107	0.999	0.985
1000	iii	99.4	0.928	0.200	0.999	0.985
1000	iv	109	0.895	0.219	0.999	0.985
2000	-	99.8	0.969	0.094	0.999	0.997
2000	i	325	0.904	0.229	0.999	0.997
2000	ii	99.7	0.962	0.125	0.999	0.997
2000	iii	98.8	0.895	0.244	0.999	0.997
2000	iv	97.6	0.867	0.255	0.999	0.997

100% / 0 nm — 90% / 10 nm

Percentage of Ground Truth / Nearest Neighbour Distance

Supplementary Figure 4: Effect of varying moving median gate size on background removal. *The 3 Ground Truths (GT) from Fig. 1 were mixed with backgrounds i-iv (Fig. 1), giving 12 datasets that were either corrected with the moving median correction or left uncorrected. To assess the effect of the moving gate size, the number of frames in the gate was varied; 50, 100, 200, 500, 1000 and 2000, the resulting images were then compared to the GT. This was carried out with* ***A)*** *backgrounds with constant intensity and* ***B)*** *backgrounds with decaying intensity. Full data in supplementary Table 6.*

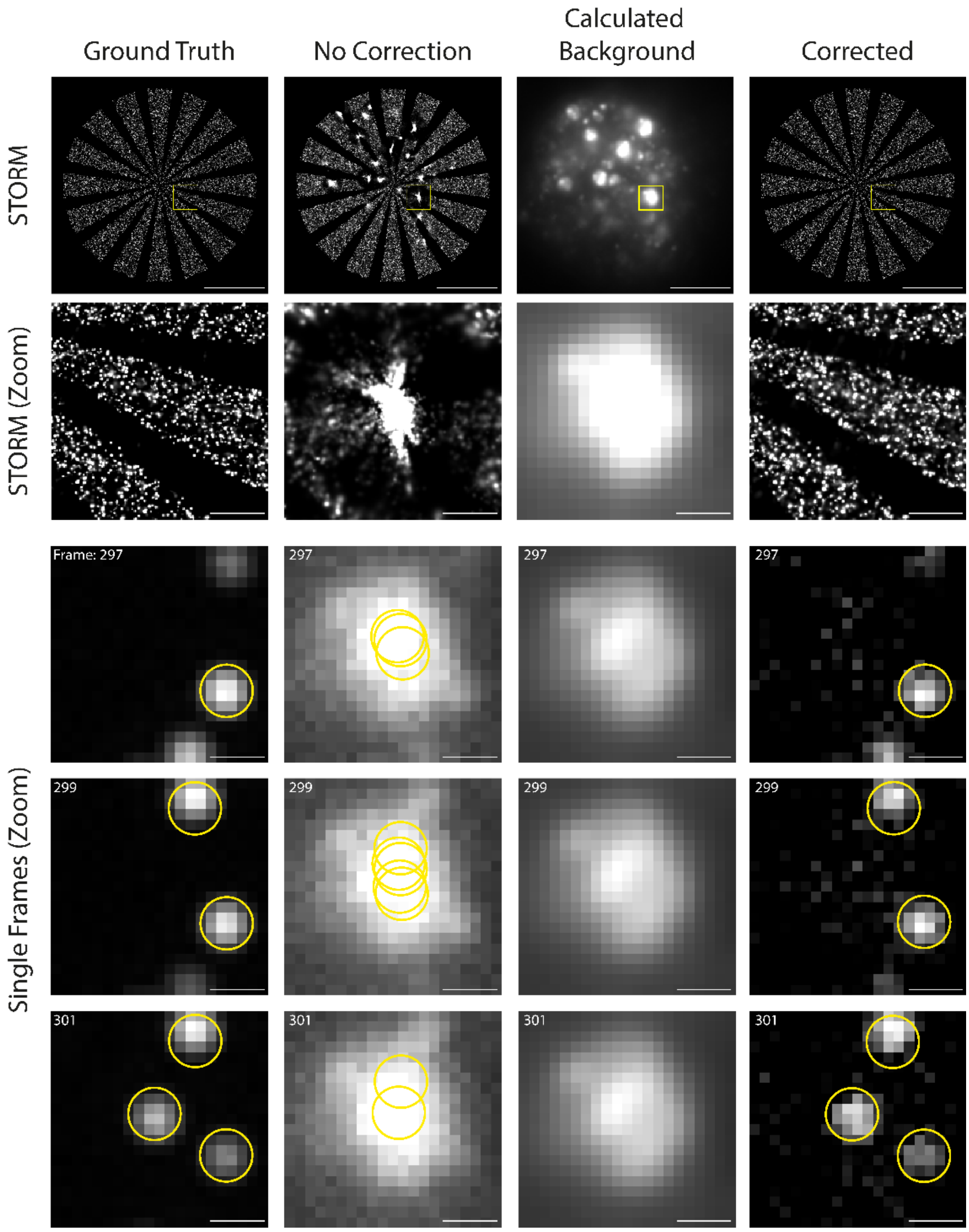

Supplementary Figure 5: Demonstrating the background calculated by a moving median filter. *An example of the Star ground truth with a zoomed region (2 x 2 µm) and 3 alternating frames from this region, with detected events circled. The ground truth was mixed with the auto-fluorescent background of a lung macrophage and the same region and frames are shown, with detected events indicated. The moving median was then used to calculate the background, this is shown in the third column and this was then subtracted to give the corrected dataset in the 4th column. Scale bars: 5 µm or 500 nm (Single frames / Zoomed Single frames).*

A

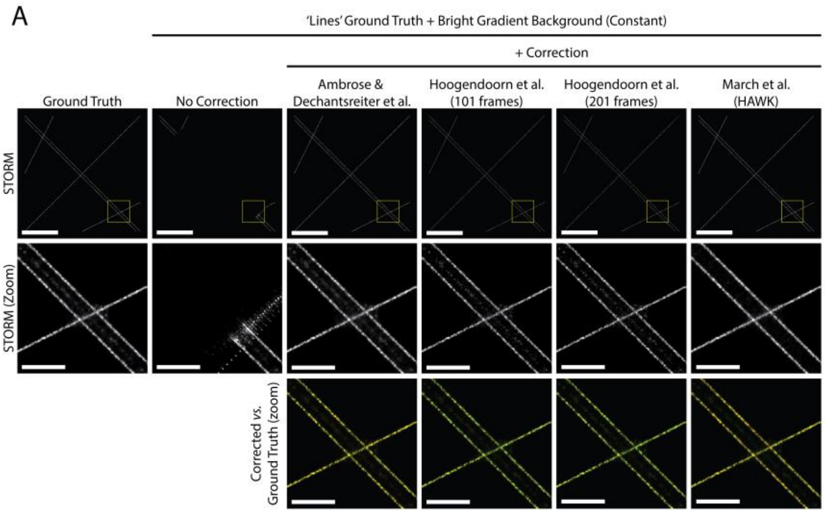

B

	% of events	CBC	NND (nm)	Manders Overlap Coefficient	Pearsons Correlation Coefficient
Ground Truth	100 ±0	1 ±0	0 ±0	1 ±0	1 ±0
No Correction	61.5 ±42.0	0.059 ±0.829	101 ±150	0.837 ±0.29	0.552 ±0.438
Ambrose & Dechantsreiter *et al.*	99.8 ±1.30	0.965 ±0.023	0.107 ±0.048	0.995 ±0.01	0.975 ±0.023
Hoogendoorn *et al.* (101 frames)	100.1 ±0.30	0.965 ±0.022	0.105 ±0.047	1.00 ±0.00	0.934 ±0.05
Hoogendoorn *et al.* (201 frames)	100.1 ±0.30	0.982 ±0.011	0.075 ±0.040	1.00 ±0.00	0.945 ±0.042
March et al. (HAWK)	362.3 ±26.1	0.912 ±0.062	0.130 ±0.036	0.999 ±0.001	0.922 ±0.069

0% / 0 nm — 10% / 1 nm

Percentage difference from Ground Truth / Nearest Neighbour Distance

C

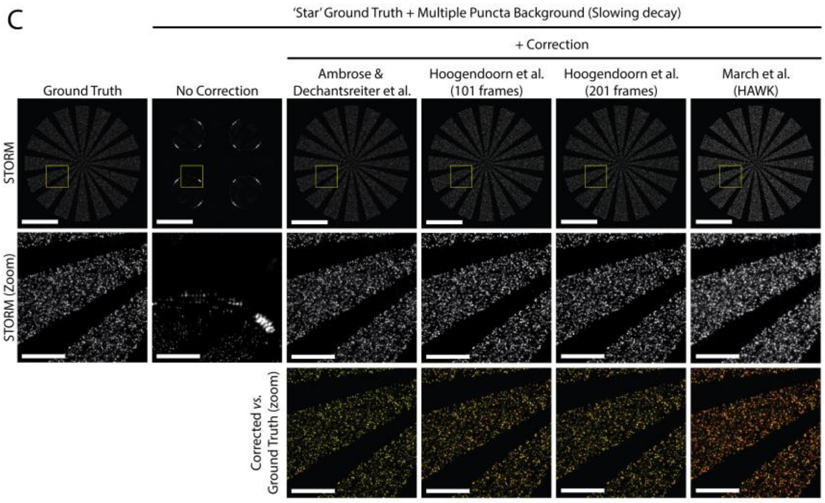

D

	% of events	CBC	NND (nm)	Manders Overlap Coefficient	Pearsons Correlation Coefficient
Ground Truth	100 ±0	1 ±0	0 ±0	1 ±0	1 ±0
No Correction	65.7 ±40.2	0.166 ±0.798	41.0 ±65.6	0.827 ±0.303	0.574 ±0.435
Ambrose & Dechantsreiter *et al.*	99.4 ±0.9	0.947 ±0.035	0.152 ±0.063	0.999 ±0.001	0.996 ±0.001
Hoogendoorn *et al.* (101 frames)	99.8 ±0.5	0.953 ±0.031	0.136 ±0.061	1.00 ±0.00	0.929 ±0.052
Hoogendoorn *et al.* (201 frames)	99.8 ±0.6	0.96 ±0.03	0.126 ±0.065	1.00 ±0.00	0.934 ±0.048
March et al. (HAWK)	362.5 ±26.1	0.912 ±0.062	0.130 ±0.036	0.996 ±0.008	0.923 ±0.069

0% / 0 nm — 10% / 1 nm

Percentage difference from Ground Truth / Nearest Neighbour Distance

E

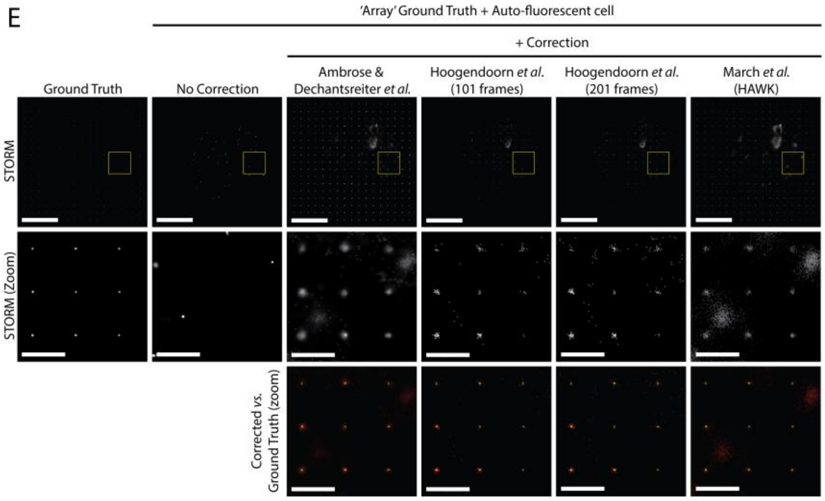

F

	% of events	CBC	NND (nm)	Manders Overlap Coefficient	Pearsons Correlation Coefficient
Ground Truth	100 ±0	1 ±0	0 ±0	1 ±0	1 ±0
No Correction	539.3 ±501.2	-0.14 ±0.429	46.1 ±42.1	0.613 ±0.35	0.252 ±0.235
Ambrose & Dechantsreiter *et al.*	94.6 ±7.2	0.954 ±0.017	0.420 ±0.310	0.918 ±0.08	0.885 ±0.068
Hoogendoorn *et al.* (101 frames)	64.6 ±32.1	0.733 ±0.311	0.438 ±1.11	0.928 ±0.15	0.736 ±0.236
Hoogendoorn *et al.* (201 frames)	66.3 ±31.7	0.752 ±0.297	0.423 ±1.08	0.915 ±0.167	0.754 ±0.232
March et al. (HAWK)	243.3 ±68.1	0.86 ±0.077	0.690 ±0.999	0.81 ±0.205	0.816 ±0.069

0% / 0 nm — 10% / 1 nm

Percentage difference from Ground Truth / Nearest Neighbour Distance

Supplementary Figure 6: Comparison to other methods of background separation. *Background was removed from datasets using the technique outlined in this manuscript (200 frame moving median) or using the Python implemented method of Hoogendoorn et al. (101 and 201 frames) or using HAWK. These 5 methods were used to correct STORM datasets and then compared to the Ground Truths. A & B) Comparison of the 5 techniques using the 12 datasets from Figure 1 (3 ground truths, each mixed with 4 backgrounds with constant intensity). A) Example images from the Ground Truth, non-corrected and 5 differently corrected datasets showing full processed STORM images (scale bar = 5 µm), zoomed*

sections and zoomed sections overlaid with the ground truth images (scale bar = 1 µm). B) Comparing the 5 techniques by assessing, relative to the ground truth, the events detected, the coordinate based colocalization, nearest neighbour distance, Manders overlap coefficient and Pearsons correlation coefficient. C & D) Comparison of the 5 techniques using the 12 datasets from Figure 3 (3 ground truths, each mixed with 4 backgrounds with slowing decaying intensity). C) Example images from the Ground Truth, non-corrected and 5 differently corrected datasets showing full processed STORM images (scale bar = 5 µm), zoomed sections and zoomed sections overlaid with the ground truth images (scale bar = 1 µm). D) Comparing the 5 techniques by assessing, relative to the ground truth, the events detected, the coordinate based colocalization, nearest neighbour distance, Manders overlap coefficient and Pearsons correlation coefficient. E & F) Comparison of the 5 techniques using the 9 datasets from Figure 4 (3 ground truths, each mixed with 3 acquired auto-fluorescent backgrounds). E) Example images from the Ground Truth, non-corrected and 5 differently corrected datasets showing full processed STORM images (scale bar = 5 µm), zoomed sections and zoomed sections overlaid with the ground truth images (scale bar = 1 µm). F) Comparing the 5 techniques by assessing, relative to the ground truth, the events detected, the coordinate based colocalization, nearest neighbour distance, Manders overlap coefficient and Pearsons correlation coefficient. Full data in supplementary Table 7.

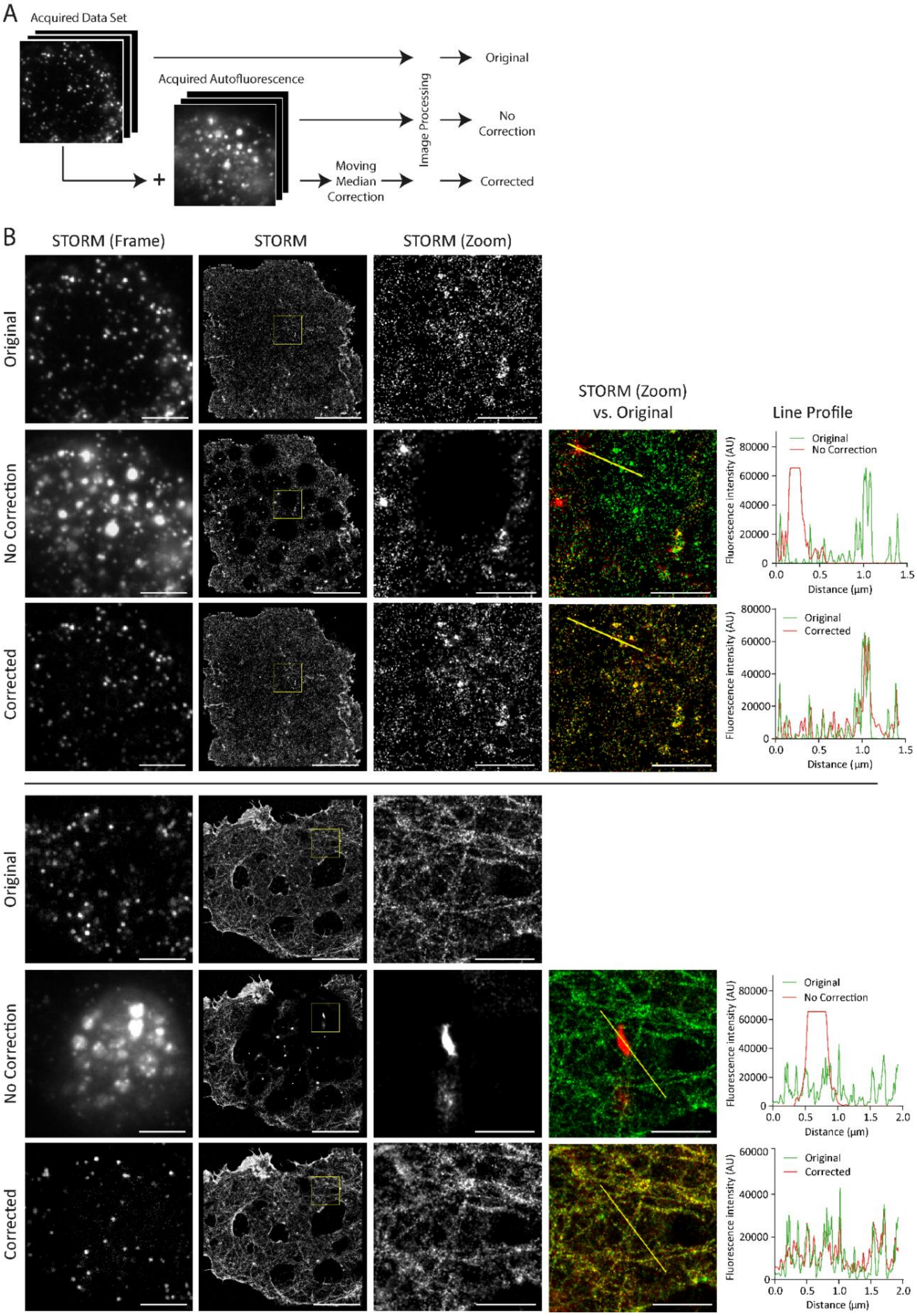

Supplementary Figure 7: Testing moving median correction by combining severe auto-fluorescence and separately acquired STORM datasets. A) *STORM datasets were acquired from stained HEK293T cells and combined with acquired datasets of auto-fluorescent lung macrophages.* ***B)*** *Auto-fluorescence from lung macrophages was combined with HEK293T cells stained for (top) MHC class I with an AF647 conjugated mAb or (bottom) filamentous actin stained with phalloidin-AF647. The data for each stain is separated into the original, cellular 'ground truth', the combined auto-fluorescence and original dataset and the combined but corrected data shown. Examples display individual frames from STORM datasets,*

super-resolution STORM images, zoomed sections, overlays and corresponding line profiles. Scale bars: 5µm and 1µm (Zoom).

Supplementary Table 1. Breakdown of data for Figure 1B

	Correction Method	Gate size	Ground Truth	Background	Background Change	Event (% of Ground Truth)	CBC	NND	Manders	Pearsons
1	Ground Truth	N/A	Array	None	N/A	100	1	0	1	1
2	Ground Truth	N/A	Lines	None	N/A	100	1	0	1	1
3	Ground Truth	N/A	Star	None	N/A	100	1	0	1	1
4	Moving Mean	200	Array	Bright gradient	Constant	100.98	0.99	0.05	1	0.999
5	Moving Mean	200	Lines	Bright gradient	Constant	95.62	0.8	0.5	0.983	0.951
6	Moving Mean	200	Star	Bright gradient	Constant	97.57723324	0.916548368	0.245936595	0.9616	0.8725
7	Moving Mean	200	Array	Dim gradient	Constant	100.97	0.99	0.05	1	0.999
8	Moving Mean	200	Lines	Dim Gradient	Constant	95.62	0.8	0.5	0.986	0.951
9	Moving Mean	200	Star	Dim gradient	Constant	97.57673017	0.916576373	0.245917437	0.9617	0.8725
10	Moving Mean	200	Array	Multiple puncta	Constant	100.97	0.99	0.05	1	0.999
11	Moving Mean	200	Lines	Multiple puncta	Constant	95.62	0.8	0.5	0.985	0.95
12	Moving Mean	200	Star	Multiple puncta	Constant	97.57522097	0.916586959	0.245935826	0.9618	0.8726
13	Moving Mean	200	Array	Uniform	Constant	100.98	0.99	0.05	1	0.999
14	Moving Mean	200	Lines	Uniform	Constant	95.62	0.8	0.5	0.986	0.951
15	Moving Mean	200	Star	Uniform	Constant	97.57572404	0.916572626	0.245890047	0.9617	0.8725
16	Moving Median	200	Array	Bright gradient	Constant	101.3808312	0.995598765	0.03925542	0.9978	0.9946
17	Moving Median	200	Lines	Bright gradient	Constant	99.8048093	0.960904035	0.14737375	0.997	0.9804
18	Moving Median	200	Star	Bright gradient	Constant	98.29240291	0.939839208	0.13572578	0.9988	0.9711
19	Moving Median	200	Array	Dim gradient	Constant	101.3808312	0.995600014	0.03924616	0.998	0.9971
20	Moving Median	200	Lines	Dim Gradient	Constant	99.80480932	0.960901078	0.14737773	0.9996	0.9962
21	Moving Median	200	Star	Dim gradient	Constant	98.29240294	0.939839934	0.13572393	0.9991	0.9932
22	Moving Median	200	Array	Multiple puncta	Constant	101.3808311	0.995599439	0.03925195	0.9623	0.9391
23	Moving Median	200	Lines	Multiple puncta	Constant	99.8048093	0.96090076	0.14737495	0.9941	0.9737
24	Moving Median	200	Star	Multiple puncta	Constant	98.29240295	0.939839994	0.13572494	0.9986	0.9727
25	Moving Median	200	Array	Uniform	Constant	101.3808311	0.995598103	0.03925564	0.9993	0.9992
26	Moving Median	200	Lines	Uniform	Constant	99.80480929	0.96090683	0.14737637	0.995	0.9641
27	Moving Median	200	Star	Uniform	Constant	98.29240293	0.939839642	0.13572927	0.9974	0.9234
28	None	N/A	Array	Bright gradient	Constant	48.69136058	-0.742565276	447.0221004	0.4946	0.8806
29	None	N/A	Lines	Bright gradient	Constant	26.17567158	-0.515516764	159.0344533	0.9047	0.8305
30	None	N/A	Star	Bright gradient	Constant	59.65327828	-0.179791219	2.99159911	0.7666	0.0106

31	None	N/A	Array	Dim gradient	Constant	99.9343711	0.864908164	0.41349224	0.9306	0.7417
32	None	N/A	Lines	Dim Gradient	Constant	99.90341083	0.808520775	0.33070241	0.9855	0.8701
33	None	N/A	Star	Dim gradient	Constant	99.55562672	0.420291176	0.81706932	0.9775	0.4132
34	None	N/A	Array	Multiple puncta	Constant	0.081379781	-1	366.5103473	0	0
35	None	N/A	Lines	Multiple puncta	Constant	0.218834893	-0.987296998	166.4261155	0.9976	-0.1097
36	None	N/A	Star	Multiple puncta	Constant	3.660195309	-0.948185589	67.77197178	0.9913	-0.0128
37	None	N/A	Array	Uniform	Constant	100.0761295	0.999993461	8.23E-08	1	1
38	None	N/A	Lines	Uniform	Constant	100.0080491	0.999622419	8.52E-04	1	1
39	None	N/A	Star	Uniform	Constant	100.5308359	0.992775277	0.00956424	0.9998	0.9998
40	Single Mean	N/A	Array	Bright gradient	Constant	101.07	0.99	0.05	1	0.999
41	Single Mean	N/A	Lines	Bright gradient	Constant	95.67	0.84	0.4	0.986	0.956
42	Single Mean	N/A	Star	Bright gradient	Constant	97.63005519	0.925983977	0.231744556	0.9625	0.8919
43	Single Mean	N/A	Array	Dim gradient	Constant	101.07	0.99	0.05	1	0.999
44	Single Mean	N/A	Lines	Dim Gradient	Constant	95.67	0.84	0.4	0.987	0.956
45	Single Mean	N/A	Star	Dim gradient	Constant	97.63307358	0.925971891	0.23170324	0.9624	0.8919
46	Single Mean	N/A	Array	Multiple puncta	Constant	101.07	0.99	0.05	1	0.999
47	Single Mean	N/A	Lines	Multiple puncta	Constant	95.67	0.84	0.4	0.988	0.957
48	Single Mean	N/A	Star	Multiple puncta	Constant	97.63156438	0.925940047	0.231730598	0.9628	0.8921
49	Single Mean	N/A	Array	Uniform	Constant	101.07	0.99	0.05	1	1
50	Single Mean	N/A	Lines	Uniform	Constant	95.67	0.84	0.4	0.986	0.956
51	Single Mean	N/A	Star	Uniform	Constant	97.63911038	0.926011119	0.231633555	0.9625	0.8921
52	Single Median	N/A	Array	Bright gradient	Constant	101.41	0.99	0.04	1	1
53	Single Median	N/A	Lines	Bright gradient	Constant	98.29	0.94	0.16	0.974	0.971
54	Single Median	N/A	Star	Bright gradient	Constant	99.67250391	0.945530575	0.17064699	0.971	0.909
55	Single Median	N/A	Array	Dim gradient	Constant	101.41	0.99	0.04	1	1
56	Single Median	N/A	Lines	Dim Gradient	Constant	98.29	0.94	0.16	0.974	0.971
57	Single Median	N/A	Star	Dim gradient	Constant	99.67250391	0.945530575	0.17064699	0.9722	0.9419
58	Single Median	N/A	Array	Multiple puncta	Constant	101.41	0.99	0.04	1	1
59	Single Median	N/A	Lines	Multiple puncta	Constant	98.29	0.94	0.16	0.974	0.971
60	Single Median	N/A	Star	Multiple puncta	Constant	99.67250391	0.945530575	0.17064699	0.9709	0.9053
61	Single Median	N/A	Array	Uniform	Constant	101.41	0.99	0.04	1	1
62	Single Median	N/A	Lines	Uniform	Constant	98.29	0.94	0.16	0.974	0.971
63	Single Median	N/A	Star	Uniform	Constant	99.67250391	0.945530575	0.17064699	0.9677	0.8575

Supplementary Table 2. Breakdown of data for Figure 2C-G

	Correction Method	Gate size	Ground Truth	Background	Background Change	Event (% of Ground Truth)	CBC	NND	Manders	Pearsons
1	Ground Truth	N/A	Array	None	N/A	100	1	0	1	1
2	Ground Truth	N/A	Lines	None	N/A	100	1	0	1	1
3	Ground Truth	N/A	Star	None	N/A	100	1	0	1	1
4	Moving Median	200	Array	Bright gradient	Constant	101.3808312	0.995598765	0.03925542	0.9978	0.9946
5	Moving Median	200	Lines	Bright gradient	Constant	99.8048093	0.960904035	0.14737375	0.997	0.9804
6	Moving Median	200	Star	Bright gradient	Constant	98.29240291	0.939839208	0.13572578	0.9988	0.9711

Supplementary Table 3. Breakdown of data for Figure 3E-I

	Correction Method	Gate size	Ground Truth	Background	Background Change	Event (% of Ground Truth)	CBC	NND	Manders	Pearsons
1	Ground Truth	N/A	Array	None	N/A	100	1	0	1	1
2	Ground Truth	N/A	Lines	None	N/A	100	1	0	1	1
3	Ground Truth	N/A	Star	None	N/A	100	1	0	1	1
4	None	N/A	Array	Dim gradient	slowing-decay	99.98162396	0.951287192	0.2439998	0.97	0.8699
5	None	N/A	Array	Bright gradient	slowing-decay	64.41078413	-0.743021153	114.2831459	0.3638	0.8936
6	None	N/A	Array	Multiple puncta	slowing-decay	3.782847242	-1	177.4177338	0	0
7	None	N/A	Array	Uniform	slowing-decay	100.0551282	0.999993543	7.04E-08	1	1
8	None	N/A	Lines	Dim Gradient	slowing-decay	99.98188958	0.89300923	0.25184573	0.995	0.9352
9	None	N/A	Lines	Bright gradient	slowing-decay	31.94637291	-0.161251001	19.51425786	0.8437	0.619
10	None	N/A	Lines	Multiple puncta	slowing-decay	2.252238656	-0.982685949	163.220224	0.9981	-0.1079
11	None	N/A	Lines	Uniform	slowing-decay	100.0035215	0.999629788	8.49E-04	1	1
12	None	N/A	Star	Dim gradient	slowing-decay	100.1865846	0.673910949	0.61474005	0.991	0.6241
13	None	N/A	Star	Bright gradient	slowing-decay	75.88743887	-0.103371908	1.3488372	0.8575	0.0343
14	None	N/A	Star	Multiple puncta	slowing-decay	8.897204141	-0.524533883	14.9989715	0.9036	0.0151
15	None	N/A	Star	Uniform	slowing-decay	100.5289467	0.99277494	0.00956559	0.9998	0.9998
16	Moving Median	200	Array	Dim gradient	slowing-decay	101.3047017	0.995491613	0.04008572	0.998	0.9974
17	Moving Median	200	Array	Bright gradient	slowing-decay	100.2388891	0.990107174	0.08557856	0.998	0.9974
18	Moving Median	200	Array	Multiple puncta	slowing-decay	100.7429186	0.984090503	0.09859768	0.9982	0.9974
19	Moving Median	200	Array	Uniform	slowing-decay	99.59047596	0.991614407	0.07522761	0.998	0.9974
20	Moving Median	200	Lines	Dim Gradient	slowing-decay	99.72582752	0.960355178	0.14912649	0.9997	0.9964
21	Moving Median	200	Lines	Bright gradient	slowing-decay	98.90834093	0.929038547	0.20718182	0.9997	0.9964
22	Moving Median	200	Lines	Multiple puncta	slowing-decay	99.12466039	0.924922726	0.20092254	0.9997	0.9964
23	Moving Median	200	Lines	Uniform	slowing-decay	98.5783278	0.90291423	0.24064752	0.9997	0.9964
24	Moving Median	200	Star	Dim gradient	slowing-decay	98.34108344	0.941800563	0.13656921	0.999	0.9942
25	Moving Median	200	Star	Bright gradient	slowing-decay	98.7754301	0.927714622	0.17409715	0.999	0.9942
26	Moving Median	200	Star	Multiple puncta	slowing-decay	98.43684612	0.922465706	0.17675811	0.999	0.9942
27	Moving Median	200	Star	Uniform	slowing-decay	99.14772727	0.890871164	0.24134485	0.999	0.9942

Supplementary Table 4. Breakdown of data for Figure 4B-F

	Correction Method	Gate size	Ground Truth	Background	Background Change	Event (% of Ground Truth)	CBC	NND	Manders	Pearsons
1	Ground Truth	N/A	Array	None	N/A	100	1	0	1	1
2	Ground Truth	N/A	Lines	None	N/A	100	1	0	1	1
3	Ground Truth	N/A	Star	None	N/A	100	1	0	1	1
4	Moving Median	200	Array	AF1	N/A	112.4169795	0.967836064	3.567437735	0.753	0.78
5	Moving Median	200	Array	AF2	N/A	92.19804163	0.952962319	7.806791457	0.954	0.91
6	Moving Median	200	Array	AF3	N/A	99.15995065	0.970729952	3.974490628	0.807	0.79
7	Moving Median	200	Lines	AF1	N/A	91.42418754	0.975831	5.358835595	0.951	0.92
8	Moving Median	200	Lines	AF2	N/A	91.4780159	0.934274	9.520801363	0.91	0.84
9	Moving Median	200	Lines	AF3	N/A	85.30536271	0.971559	5.943847742	0.964	0.91
10	Moving Median	200	Star	AF1	N/A	95.12860381	0.94554	0.452765083	0.999	0.95
11	Moving Median	200	Star	AF2	N/A	90.29833178	0.926138	0.654874995	0.998	0.87
12	Moving Median	200	Star	AF3	N/A	93.67981283	0.942391	0.505012934	0.93	0.999
13	Uncorrected	N/A	Array	AF1	N/A	1349.541911	-0.270175454	30.39996275	0.813	0.04
14	Uncorrected	N/A	Array	AF2	N/A	1352.117187	-0.422183021	36.52383015	0.079	0
15	Uncorrected	N/A	Array	AF3	N/A	986.6484656	-0.331560174	34.26424951	0.815	0.04
16	Uncorrected	N/A	Lines	AF1	N/A	294.8254352	-0.40392156	86.85018595	0.488	0.19
17	Uncorrected	N/A	Lines	AF2	N/A	262.4489385	-0.666920492	121.2427485	0.217	0.02
18	Uncorrected	N/A	Lines	AF3	N/A	224.6020726	-0.491871529	96.66454085	0.19	0.465
19	Uncorrected	N/A	Star	AF1	N/A	131.4187108	0.505823463	2.29721111	0.976	0.62
20	Uncorrected	N/A	Star	AF2	N/A	130.8400663	0.306816908	3.86101494	0.963	0.32
21	Uncorrected	N/A	Star	AF3	N/A	121.5192688	0.516967855	2.532552961	0.974	0.57

Supplementary Table 5. Breakdown of data for Figure S3B

	Correction Method	Gate size	Ground Truth	Background	Background Change	Event (% of Ground Truth)	CBC	NND	Manders	Pearsons
1	Ground Truth	N/A	Array	None	N/A	100	1	0	1	1
2	Ground Truth	N/A	Lines	None	N/A	100	1	0	1	1
3	Ground Truth	N/A	Star	None	N/A	100	1	0	1	1
4	Single Median	N/A	Array	None	N/A	101.41	0.997	0.142	0.999	1
5	Single Median	N/A	Lines	None	N/A	100.74	0.985	0.305	1	0.99
6	Single Median	N/A	Star	None	N/A	99.85	0.917	0.399	0.999	0.98
7	Single Mean	N/A	Array	None	N/A	101.07	0.997	0.145	0.999	1
8	Single Mean	N/A	Lines	None	N/A	98.23	0.977	0.421	0.999	0.93
9	Single Mean	N/A	Star	None	N/A	96.18	0.888	0.68	0.998	0.76
10	Moving Median	200	Array	None	N/A	101.41	1	0	1	1
11	Moving Median	200	Lines	None	N/A	100.74	0.985	0.306	0.999	0.99
12	Moving Median	200	Star	None	N/A	98.29	0.97	0.5	1	1
13	Moving Mean	200	Array	None	N/A	100.97	0.997	0.145	0.999	1
14	Moving Mean	200	Lines	None	N/A	98.18	0.977	0.437	0.999	0.93
15	Moving Mean	200	Star	None	N/A	96.15	0.881	0.754	0.998	0.73

Supplementary Table 6 that gives a breakdown of the data from Fig. S4 has been uploaded as a separate .csv file.

Supplementary Table 7 that gives a breakdown of the data from Fig. S6 has been uploaded as a separate .csv file.
